# Making apples from oranges: Comparing noncollapsible effect estimators and their standard errors after adjustment for different covariate sets

**DOI:** 10.1002/bimj.201900297

**Published:** 2020-12-14

**Authors:** Rhian Daniel, Jingjing Zhang, Daniel Farewell

**Affiliations:** ^1^ Division of Population Medicine Cardiff University Cardiff UK

**Keywords:** covariate adjustment, Cox proportional hazards regression, logistic regression, noncollapsibility

## Abstract

We revisit the well‐known but often misunderstood issue of (non)collapsibility of effect measures in regression models for binary and time‐to‐event outcomes. We describe an existing simple but largely ignored procedure for marginalizing estimates of conditional odds ratios and propose a similar procedure for marginalizing estimates of conditional hazard ratios (allowing for right censoring), demonstrating its performance in simulation studies and in a reanalysis of data from a small randomized trial in primary biliary cirrhosis patients. In addition, we aim to provide an educational summary of issues surrounding (non)collapsibility from a causal inference perspective and to promote the idea that the words *conditional* and *adjusted* (likewise *marginal* and *unadjusted*) should not be used interchangeably.

## 1 INTRODUCTION

### 1.1 Noncollapsibility: An overview

It is well known that two of the statistical models most often used in medical research, namely, logistic regression and Cox proportional hazards (PH) regression, involve parameters of interest that are *noncollapsible*. Even in an ideal randomized controlled trial (RCT) (i.e., no dropout, non‐adherence or other complicating structural features) with a binary or right‐censored time‐to‐event outcome, no matter how large the sample size, the odds ratio or hazard ratio comparing treated and untreated individuals will change upon including a baseline covariate in the model, whenever that covariate is associated with the outcome. That is, even when there is no confounding, whether or not we include a covariate in our model matters for the magnitude of our treatment effect, whenever that covariate is predictive of the outcome. Conditioning on a covariate changes the very nature of the treatment effect we are estimating. This difference (between a conditional and marginal odds/hazard ratio) is not explained by sampling variation, and is what is referred to when it is said that odds/hazard ratios are *noncollapsible*.

**FIGURE 1:**
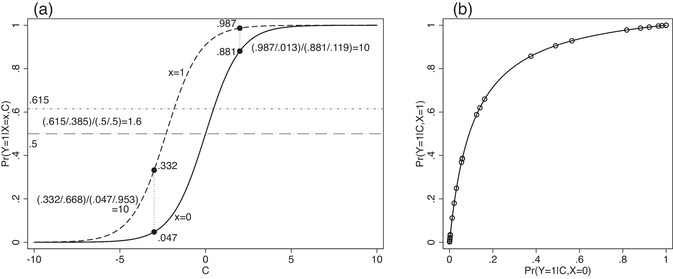
(a) A simple logistic regression model, and (b) the relationship between the conditional probability of Y=1 given C between the two treatment groups as implied by the model in (a). Thirty randomly chosen values of C∼U[−10,10] give rise to the superimposed scatter plot in (b)

Consider a simple logistic regression model with a covariate C uniformally distributed between −10 and 10, a randomized binary treatment X independent of C with Pr(X=1)=0.5 and a binary outcome Y following a logistic regression model given X and C: logit{Pr(Y=1|X,C)}=log(10)·X+C so that the conditional odds ratio comparing treated and untreated, conditional on C=c, is 10, no matter the value of c. Figure 1(a) shows the conditional probability of Y=1 as a function of C for the treated (dashed line) and untreated (solid line) groups separately.

For illustration, we show that at C=−3, the probability of Y=1 in the two groups is 0.332 and 0.047, respectively, and the ratio of the corresponding odds, $\frac{0.332}{0.668}/\frac{0.047}{0.953}$, is 10. Similarly, at C=2, the probability of Y=1 in the two groups is 0.987 and 0.881, respectively, and the ratio of the corresponding odds, $\frac{0.987}{0.013}/\frac{0.881}{0.118}$, is again 10. This emphasizes that the odds ratio of 10 corresponds to a *vertical* comparison of the two sigmoid curves in Figure 1 at any point along the C‐axis. Now suppose we compare the marginal odds of Y=1 between the two groups. This amounts to first averaging the two sigmoid curves over the distribution of C and then comparing the resulting odds. The two dot–dash lines show these two averages: They can be thought of as the averages of the y‐axis values of each curve, averaged according to the distribution of C, the x‐axis. The ratio of the odds corresponding to the dot–dash lines is approximately 1.6, which is much less than 10.

The key to understanding the difference between the conditional and marginal odds ratio is contained in the plot of Pr(Y=1|X=1,C) against Pr(Y=1|X=0,C) in Figure 1(b), inspired by the plots in Neuhaus and Jewell (1993). In our example, the function mapping Pr(Y=1|X=0,C) to Pr(Y=1|X=1,C) is

$$g\left(x\right)=\frac{\exp\left\{logit(x)+log(10)\right\}}{1+\exp\left\{logit(x)+log(10)\right\}}=\frac{10x}{1+9x}.$$

This is a nonlinear function of x; moreover it is a concave function for the relevant choice of coefficient.

More generally, if f(·) is any link function (such as identity, log, logit), and ν is the conditional (on C) association between X and Y on the scale of the linear predictor, then the function that maps Pr(Y=1|X=0,C) to Pr(Y=1|X=1,C) governs (non)collapsibility and is given by

$$g_{\nu }\left(\cdot \right)=f^{-1}\left\{f(\cdot )+\nu \right\},$$

which we call the *characteristic collapsibility function*, or CCF. The CCF first applies the link function—to convert the probability (in the untreated, as a function of C) to the scale of the linear predictor—then adds the conditional association (ν) on the scale of the linear predictor, and finally applies the inverse of the link function, returning to the probability scale. As shown by Neuhaus and Jewell (1993), the collapsibility or otherwise of effect measures is inherently linked to this change (and reverse‐change) of scale, and is determined by the nature of the CCF, as we review in detail in Appendix A.1. Note that this discussion is predicated on the (strong) assumption that the conditional association between X and Y, conditional on C=c, does not depend on c. For more general discussions of noncollapsibility, including in the presence of effect measure modification, see Greenland and Pearl (2011) and the many references therein.

**FIGURE 2:**
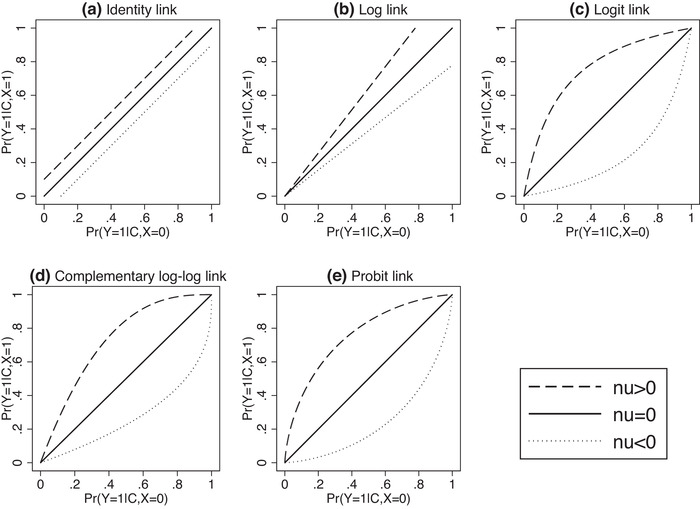
The (non)collapsibility of common effect measures for binary outcomes as determined by the concavity, convexity, or linearity of the characteristic collapsibility function (CCF) $g_{\nu }\left(\cdot \right)=f^{-1}\left\{f\left(\cdot \right)+\nu \right\}$, where f is the link function and ν is the conditional effect measure. f(p)=log{p/(1−p)} for the logit link, f(p)=log(−log(1−p)) for the complementary log‐log link, and $f\left(p\right)=\Phi^{-1}\left(p\right)$, where Φ(·) is the CDF of the standard normal distribution, for the probit link

Briefly, Pr(Y=1|X=x) is obtained from Pr(Y=1|X=x,C) (x=0,1) by averaging over C, and Pr(Y=1|X=1,C) is related to Pr(Y=1|X=0,C) via the CCF $g_{\nu }$, and these two steps (applying $g_{\nu }$ and averaging) define the relationship between the marginal and conditional effects. Jensen's inequality implies that the two steps are interchangeable if and only if the CCF is linear, but that changing the order leads to an increase or a decrease when the CCF is convex or concave, respectively. For the identity and log links, the CCF is linear, but for all other common link functions, the CCF is concave for positive ν, convex for negative ν, and linear when ν=0. This explains both the discrepancy between conditional and marginal effects referred to as noncollapsibility and, moreover, the attenuation of the marginal effect relative to the conditional effect. Figure 2 illustrates the CCF for a variety of link functions and values of ν. Note that the identity and log link functions correspond to effect measures that represent risk differences and (log) risk ratios, respectively, and both these effect measures are known to be collapsible, illustrated by the linearity of their CCFs.

The scatter plot superimposed on the curve in Figure 1(b) shows the values of Pr(Y=1|X=1,C) and Pr(Y=1|X=0,C) for 30 randomly selected values of C. This illustrates the relevance of the strength of the conditional association between C and Y given X on the extent of noncollapsibility. If this association were weaker (equivalently, if the variance of C were smaller), the points would cluster more closely together, and the extent of nonlinearity relevant to the application of Jensen's inequality would decrease. If there is no association between C and Y conditional on X, there is only one relevant point, the expectation step (over C) can be removed, and all measures are collapsible.

To summarize, the noncollapsibility of the odds ratio is a consequence of the logit link function, which implies a nonlinear CCF. A linear CCF is implied not only by a linear link function (such as the identity), but also by the log link function; thus, risk differences and ratios are collapsible. In general, however, as we see in Figure 2, most commonly used link functions for binary outcomes imply a nonlinear CCF, and hence noncollapsible effect measures. It is generally seen as a desirable feature that the curves in Figure 2(c)–(e) coincide at 0 and 1. This is what prevents such models from predicting probabilities outside the range [0,1], in contrast to (a) and (b). Noncollapsibility is thus an inevitable consequence of the “bending” of the function that must take place in order to respect both the upper and lower boundaries of probabilities.

In models for time‐to‐event outcomes, the probability (risk) above is replaced by a rate or hazard (where we use *rate* for the discrete‐time version and *hazard* for the continuous‐time version; see Appendix A.2 and Section 2 for more details). In contrast to binary outcomes (where the logit link, via logistic regression, is the usual choice), the most commonly used functions for mapping rates/hazards to linear predictors in time‐to‐event models are the log link (e.g., in the Cox PH model) and the identity link (e.g., in the Aalen additive hazards model). It might be tempting to think, therefore, that noncollapsibility is not an issue for rate/hazard differences/ratios.

Sjölander, Dahlqwist, and Zetterqvist (2015) explain why this reasoning is faulty. Crucially, rates and hazards are based on conditional probabilities (conditional on survival), and these cannot be averaged over C as above (see Appendix A.2). An additional step that removes this conditioning on survival converts a rate/hazard model into a risk model (the probability of an event before time t), but this step alters the link function. For rates (arising from survival models in discrete time), the corresponding risk link functions imply a nonlinear CCF even when the rate link function is either the identity or the log link; thus, rate differences and rate ratios are both noncollapsible.

As time is subdivided into more intervals, and the probability of the event in any given interval decreases (and the rate becomes a hazard), the risk link function corresponding to an additive hazards model is the (complementary) log link and that corresponding to a PH model is the complementary log–log link. Thus, hazard differences (unlike rate differences) are collapsible, but hazard ratios are not.

**FIGURE 3:**
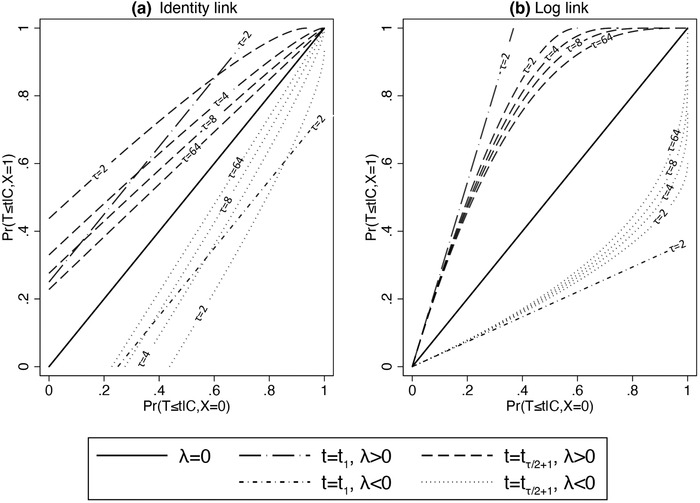
The CCF implied by discrete‐time rate models with (a) an identity link and (b) a log link, for both the first time‐interval, and a subsequent time‐interval, as well as for different values of τ, the total number of time intervals, and for different treatment effect values (λ) on the scale of the linear predictor. As τ→∞, the discrete‐time rate model becomes a continuous‐time hazard model

This is illustrated in Figure 3, where the CCFs (as calculated from the implied risk model) corresponding to the identity and log link functions for the rate model are shown. These are shown for the rate during the first (of two) time intervals, as well as for a time interval half‐way through follow‐up, with follow‐up divided into increasingly many intervals. Note that the CCF for both link functions is linear for the first, but not for subsequent, time intervals: this is since the conditioning on survival is relevant only from the second interval onward. Note also that the CCFs in (a) become closer to linear as τ increases, but that the same is not the case in (b). Finally, note that Figure 3(b), as τ increases, is the same shape as Figure 1(d), and Figure 3(a), as τ increases, is a reflection of Figure 1(b): PH models correspond to probability models with a complementary log–log link, and additive hazards models correspond to probability models with a (complementary) log link.

To summarize, Sjölander et al. (2015) point out that “the mechanism that induces noncollapsibility is quite different for the rate difference [and rate ratio] than for the odds ratio, since the latter is noncollapsible due to the nonlinearity of the logistic transformation [whereas] the mechanism that makes the rate difference noncollapsible [is] conditioning on past survival.” However, by converting the probabilities that condition on past survival to ones that do not, a new link function is obtained, relevant to forming the CCF. This leads us back to a unified view of noncollapsibility in models for binary and time‐to‐event outcomes.

### 1.2 Noncollapsibility in observational studies and mediation analysis

In non‐randomized studies analyzed using models with parameters that suffer from noncollapsibility, we should be mindful that when adding or removing potential confounders from our model, any changes we see in our exposure effect estimate will be due to a combination of noncollapsibility and confounding (as well as finite sample variation), which complicates the use of change‐in‐estimate procedures for deciding when a covariate is a confounder (Greenland & Robins, 1986; Greenland, 1987; Greenland, Robins, & Pearl, 1999; Martinussen & Vansteelandt, 2013; Miettinen & Cook, 1981; Pang, Kaufman, & Platt, 2013; Vansteelandt, Bekaert, & Claeskens, 2012; Wickramaratne & Holford, 1987). Similarly, when using traditional methods for mediation analysis, we should be mindful that when including/excluding potential mediators in/from our model, any changes we see in our exposure effect estimate will be due to a combination of noncollapsibility and mediation (as well as finite sample variation), one of many criticisms aimed at the so‐called “difference method” for mediation analysis (Lange & Hansen, 2011; VanderWeele & Vansteelandt, 2009).

### 1.3 Covariate adjustment in RCTs: An often confusingly presented issue

When considering baseline covariate adjustment in RCTs analyzed using models that suffer from noncollapsibility, we are often warned to expect a seemingly paradoxical “trade‐off,” namely, that covariate adjustment leads to *increased* power to detect a non‐null treatment effect, but *decreased* precision for the treatment effect estimator (Begg & Lagakos, 1993; Burgess, 2017; Ford, Norrie, & Ahmadi, 1995; Gail, Tan, & Piantadosi, 1988; Karrison & Kocherginsky, 2018; Robinson & Jewell, 1991). The “paradox” is resolved upon realizing that the precision comparison is made for estimators of different estimands; on the other hand, since the conditional and marginal estimands share the same null, the power comparison is meaningful (Gail et al., 1988). Conditional and marginal odds ratios (likewise hazard ratios) are like apples and oranges. It is true that, in the absence of confounding etc., the standard error of the maximum likelihood estimator (MLE) of a conditional odds ratio is at least as large as the standard error of the corresponding MLE of a marginal odds ratio (similarly for hazard ratios) (Ford et al., 1995; Robinson & Jewell, 1991). This statement is arguably irrelevant, however, since it says that the standard error of an estimator of an orange is at least as large as the standard error of an estimator of an apple. The property regarding the comparative magnitude of the standard error of the conditional and marginal estimands is accompanied by the property that the conditional estimand is at least as far from the null as the marginal estimand, but with the estimand increasing faster than the standard error, leading to the result on power.

### 1.4 A pervasive issue

Noncollapsibility complicates many important areas of applied statistics. As already alluded to, in RCTs, it complicates the comparison of estimates and their standard errors when different baseline covariate adjustment sets have been used, and thus also comparisons of findings between RCTs. In observational studies, it further complicates procedures for confounder selection, for method choice and comparison (since, e.g., estimands conditional on the propensity score differ from estimands conditional on the covariates used in that propensity score; Austin, 2013), and for assessing mediation. In meta‐analysis, naïvely combining estimators of noncollapsible estimands from different studies that are conditional on different covariate sets is clearly problematic (Hauck, Anderson, & Marcus, 1998), likewise when attempting to triangulate evidence from a range of study designs (Lawlor, Tilling, & Davey Smith, 2016).

### 1.5 This paper

A simple (but surprisingly often ignored) method exists for turning estimates of conditional odds ratios into estimates of the marginal odds ratio (Zhang, 2008). In the light of all the issues listed above, this is clearly a useful procedure for allowing like‐with‐like comparisons, even if the marginal estimand is not ultimately the scientific focus. A corresponding method for marginalizing estimates of conditional hazard ratios follows in principle from theoretical results in Struthers and Kalbfleisch (1986). Stuthers' and Kalbfleisch's result, however, involves intractable integrals in realistic scenarios with several covariates and censoring. In this paper, we propose a procedure (using estimation by Monte Carlo simulation) for implementing Stuthers' and Kalbfleisch's marginalization in practice.

As well as this, we hope to provide an educational summary of issues surrounding noncollapsibility from a causal inference perspective and to promote the idea that the words “conditional” and “adjusted” should not be used interchangeably.

Noncollapsibility does not simply refer to a binary distinction between a marginal and a conditional estimand; conditioning on each different covariate set leads to a different conditional estimand, and if we were considering alternative methods, then adjustment for the propensity score, say, leads to yet a different conditional estimand, namely one that is conditional on the propensity score. The suitable analogy is not one of apples and oranges, therefore, but rather an entire greengrocery. We will focus, however, on a marginal versus conditional dichotomy, where the conditional estimand is conditional on a set of covariates C. Any procedure for turning an estimator of a conditional estimand into an estimator of a marginal estimand can equally be used to turn an estimator of an estimand conditional on C into and estimator of an estimand conditional on V⊂C. We mention this here so as not to alienate a reader for whom a fully marginal estimand is rarely of interest.

A recent literature has emerged discussing problematic aspects of the interpretation (particularly the causal interpretation) of hazard ratios, referring both to the noncollapsibility and the in‐built selection bias of the hazard ratio (Aalen, Cook, & Røysland, 2015; Hernán, 2010; Martinussen, Vansteelandt, & Andersen, 2018), which, as Sjölander et al. (2015) show, are two sides of the same coin. Related to this (see Section 2) is the concern that if both a conditional and a marginal Cox PH model are simultaneously considered, then, in general, the PH assumption holds for at most one of them. This makes defining and interpreting a single marginal causal HR estimand somewhat problematic when a conditional PH model is assumed. Briefly, the issue with selection bias is that, even in an ideal RCT, at any time t after the start of the trial, the hazard ratio compares two groups of patients with potentially different characteristics, namely, those who would survive to time t if assigned to one arm versus those who would survive to time t if assigned to the other arm. In a heterogeneous population under a non‐null treatment effect, the time‐t survivors in the two arms differ systematically, despite being exchangeable at baseline due to randomization. This makes the usual interpretation of a hazard ratio coupled with the strongest (counterfactual) interpretation of a causal effect difficult to combine, although as Martinussen et al. (2018) point out, the alternative interpretation of a constant hazard ratio as the ratio of the logarithms of the survivor functions in the two arms is, albeit less natural, better suited to a strong causal interpretation. We will ignore the important selection bias issue in this paper, taking instead the definition of a causal marginal hazard ratio simply to be the marginal hazard ratio that would be estimated in an infinitely large RCT, in a spirit similar to that advocated by Hernán and Robins (2016). Whether one then shares the views of Hernán (2010) and Aalen et al. or those of Martinussen et al. (or any other view) will not concern us. This “hypothetical RCT” definition also helps us to deal with the first concern, although with an important caveat that the censoring mechanism in operation in the hypothetical RCT then plays a role in the estimand definition, as we will discuss later. Note that alternative approaches are available that avoid these problems (as well as, in the latter two references, relying less heavily on parametric assumptions), such as methods based on estimating differences in hazards, survival probabilities, or mean restricted survival times (Aalen, 1989; Royston & Parmar, 2013; Zhang & Schaubel, 2012a, 2012b).

The relative scientific relevance of conditional and marginal estimands in different contexts, with implications for external validity and clinical decision making, has also been widely discussed (Harrell & Slaughter, 2019; Huitfeldt, Goldstein, & Swanson, 2018; Lindsey & Lambert, 1998; Lee & Nelder, 2004). Again, while acknowledging the importance of such considerations, we mostly sideline them here, focusing instead on how to estimate marginal odds and hazard ratios using covariate adjustment, irrespective of whether or not this is always a useful parameter with a straightforward interpretation.

The article is organized as follows. In Section 2, we put the informal discussion of noncollapsibility above on a more formal mathematical footing using the notation of potential outcomes from the causal inference framework. We clarify the distinction between associational and causal models/estimands, between marginal and conditional estimands, and between adjusted and unadjusted analyses. In much of applied statistics, “condition” and “adjust” are used interchangeably, but we contend that this should not be the case. In Section 3, we describe the proposal made by Zhang (2008) for marginalizing estimates of conditional odds ratios, before, in Section 4, describing our corresponding proposal for the practical implementation of the result in Struthers and Kalbfleisch (1986) for hazard ratios. We demonstrate the performance of our proposal in a simulation study (Section 5) and by reanalyzing data from a small RCT in primary biliary cirrhosis (PBC) patients (Section 6), before concluding with a discussion (Section 7).

## 2 NOTATION AND FRAMEWORK

Let X denote a binary exposure or treatment variable (X=1 for the exposed/treated, and X=0 for the unexposed/untreated), let Y denote the binary or right‐censored time‐to‐event outcome, according to the context, and C a set of covariates. When Y is a right‐censored time‐to‐event outcome, let D denote the event indicator, which is 1 when Y is an event time, and 0 when Y is a censoring time; we assume independent censoring throughout (Andersen, Borgan, Gill, & Keiding, 2012). The set C is likely to include potential confounders in an observational study, or simply baseline covariates in an RCT. We start by clarifying a few important distinctions: associational versus causal models, marginal versus conditional estimands, and unadjusted versus adjusted analyses.

Nothing in this section is novel, and, for example, Parts I and II of Hernán and Robins (2020) could be viewed as a reference for the whole section. Note that we express the causal aspects of our discussion using potential outcomes, but we could alternatively use counterfactual‐free notions of causality such as the do‐notation (Pearl, 2009) or other ways of expressing hypothetical interventions (Dawid, 2000).

### 2.1 Associational and causal models

Consider the following simple logistic regression model for a binary Y:

(1) $$\mathrm{logit}\left\{\mathrm{Pr}\left(Y=1\left|X=x\right)\right)\right\}:=\log\left\{\frac{\mathrm{Pr}\left(Y=1\left|X=x\right)\right)}{1-\mathrm{Pr}\left(Y=1\left|X=x\right)\right)}\right\}=\alpha +\beta x.$$

Since X is binary, this is a saturated model and is hence necessarily correctly specified as long as our assumption concerning how our data are sampled from this distribution (e.g., i.i.d.) holds. It simply says that α is the log odds of the outcome in the unexposed, and β is the log odds ratio comparing the log odds of the outcome in the exposed versus the unexposed. This is an associational model comparing the distribution of Y between exposed and unexposed individuals.

If, instead, Y is a right‐censored time‐to‐event outcome with event indicator D and corresponding hypothetical uncensored time‐to‐event outcome T, then the relative risk model (often called the Cox PH model) is

(2) $$h\left(t|x\right):=\lim_{\Delta t\to 0}\frac{Pr\left(t\leq T<t+\Delta t|X=x,T\geq t\right)}{\Delta t}=h_{0}\left(t\right)\exp\left(\psi x\right),$$

where $h_{0}\left(t\right)$, the baseline hazard function, is left unspecified (except that it must be positive). Note that, in contrast to (1), model (2) is not necessarily correctly specified, since it makes the PH assumption, namely that

$$\frac{h(t|1)}{h(t|0)}=\exp\left(\psi \right)$$

does not vary with t. Under this PH assumption, however, the interpretation of ψ in (2) is, analogously to the interpretation of β in (1), associational. It is, at each t, the log hazard ratio comparing the log hazard function for the outcome at time t in the exposed versus the unexposed, assumed to be constant over t.

Now suppose that $Y_{1}$ is the potential outcome (binary or right‐censored time‐to‐event) if, possibly counter to fact, this individual were set to be exposed, and $Y_{0}$ the corresponding potential outcome if this individual were set to be unexposed. Then, for a binary outcome, we might write down the following (saturated) logistic regression model:

(3) $$logit\left\{Pr\left(Y_{x}=1\right)\right\}=\theta +\phi x,$$

which is now a *causal* model, since it describes not the distribution of Y and X in the real world, but the distribution of Y in a hypothetical world in which we have intervened on X. The parameter ϕ is now the *causal* log odds ratio since it compares the log odds of the outcome if everyone were exposed versus if everyone were unexposed. In an ideal RCT, β=ϕ, but in an observational study, the exposure–outcome relationship will be confounded so that β≠ϕ. However, under an assumption that the covariates C are sufficient to control for confounding, technically that

(4) $$Y_{x}⊥⊥X\left|C\right),\;\;\;\;\;\;x=0,1$$

then, in addition to the technical assumption of counterfactual consistency (Cole & Frangakis, 2009; Hernan & Taubman, 2008; Pearl, 2010; VanderWeele, 2009) typically made in these situations, we have that the associational log odds ratio ν in the model

(5) $$\mathrm{logit}\left\{\mathrm{Pr}\left(Y=1\left|X=x,C=c\right)\right)\right\}=\mu +\nu x+\gamma^{T}c$$

is equal to the causal log odds ratio ζ in the model

(6) $$\mathrm{logit}\left\{\mathrm{Pr}\left(Y_{x}=1\left|C=c\right)\right)\right\}=\eta +\zeta x+\tau^{T}c.$$

This equivalence additionally requires that the functional forms of models (5) and (6) be correctly specified, an assertion which is not trivial now that we have included C in the model. In particular, these models do not include product terms between x and c, an assumption we make throughout. See Huitfeldt (2019) for a discussion of noncollapsibility that does not make this assumption.

Likewise, for a right‐censored time‐to‐event outcome, where the corresponding uncensored potential event times under the two treatments are $T_{0}$ and $T_{1}$, we might consider the model:

(7) $$h_{x}^{*}\left(t\right):=\lim_{\Delta t\to 0}\frac{Pr\left(t\leq T_{x}<t+\Delta t|T_{x}\geq t\right)}{\Delta t}=h_{0}^{*}\left(t\right)\exp\left(\kappa x\right),$$

where κ is the causal log hazard ratio, in the sense discussed in Section 1.5. In an ideal RCT, ψ=κ, but in an observational study, confounding of the exposure–outcome relationship will render ψ≠κ. Under the aforementioned assumptions of counterfactual consistency and no unmeasured confounding, we have that the associational log hazard ratio λ in the model

(8) $$\tilde{h}\left(t|x,c\right):=\lim_{\Delta t\to 0}\frac{Pr\left(t\leq T<t+\Delta t|X=x,C=c,T\geq t\right)}{\Delta t}={\tilde{h}}_{0}\left(t\right)\exp\left(\lambda x+\rho^{T}c\right),$$

is equal to the causal log hazard ratio ω in the model

(9) $${\tilde{h}}_{x}^{*}\left(t|c\right):=\lim_{\Delta t\to 0}\frac{Pr\left(t\leq T_{x}<t+\Delta t|C=c,T_{x}\geq t\right)}{\Delta t}={\tilde{h}}_{0}^{*}\left(t\right)\exp\left(\omega x+\xi^{T}c\right).$$

This equivalence additionally requires that the functional forms of models (8) and (9) be correctly specified, that is, that the PH assumption holds given C. Of note is the fact that, in general, if the PH assumption holds marginally, it does *not* hold conditional on C and *vice versa*, meaning that, in general, at most one of (2) and (8) can be correctly specified, and at most one of (7) and (9) (Ford et al., 1995). See Hougaard (1986) for special situations in which a PH model could hold both conditionally and marginally.

### 2.2 Marginal and conditional estimands

As we have stated, β, ν, ψ, and λ in (1), (5), (2), and (8), respectively, are associational parameters in associational models, whereas ϕ, ζ, κ, and ω in (3), (6), (7), and (9), respectively, are causal parameters in causal models. Another important distinction is that β, ϕ, ψ, and κ are marginal estimands, whereas ν, ζ, λ, and ω are conditional estimands, in particular conditional on C. For example, the interpretation of ζ, where

(10) $$\zeta =\log\left\{\frac{\mathrm{Pr}\left(Y_{1}=1\left|C=c\right)\right)}{1-\mathrm{Pr}\left(Y_{1}=1\left|C=c\right)\right)}\right\}-\log\left\{\frac{\mathrm{Pr}\left(Y_{0}=1\left|C=c\right)\right)}{1-\mathrm{Pr}\left(Y_{0}=1\left|C=c\right)\right)}\right\}$$

is as the true difference in log odds, for a subgroup of the population with level c of the covariates, between setting everyone's exposure to 1 versus 0. This is assumed (according to model (6)) to be a constant across values of c, but this assumption could easily be relaxed. This is a conditional interpretation.

For the causal estimand, ϕ, on the other hand, the interpretation is marginal: it is the difference in the true population log odds between setting everyone's exposure to 1 versus 0.

Even though both ϕ and ζ have a causal interpretation (neither is confounded), the right‐hand side of (10) is assumed constant across levels of c (no effect modification), and both are true population parameter values (sampling error is irrelevant), they are in general not the same: this is because the odds ratio is noncollapsible. There are two situations in which ϕ=ζ: (1) when τ=0, that is, when covariates and outcome are conditionally independent given exposure, and (2) when ζ=0, that is, when exposure and outcome are conditionally independent given covariates (in which case there is no effect of exposure on outcome, and ϕ=0 also). In all other situations, ϕ is closer to zero than ζ; that is, |ϕ|<|ζ|. For completeness, a proof of this well‐known result (Neuhaus & Jewell, 1993; Samuels, 1981) is given in Appendix A.1.

Likewise, even though both κ and ω have a causal interpretation in the sense discussed in Section 1.5 and both are true population parameter values, they are not the same: the hazard ratio is also noncollapsible, with the marginal estimand κ again closer to the null except when either the covariates are conditionally independent of the outcome given exposure or the exposure is conditionally independent of the outcome given covariates, and in this latter case κ=ω=0. For completeness, a proof of this well‐known result (Struthers & Kalbfleisch, 1986) is given in Appendix A.2.

### 2.3 Unadjusted and adjusted analyses

Unadjusted is often used as if synonymous with marginal, and adjusted synonymous with conditional. This would be sensible if we only had in mind the associational parameters. The marginal estimands β and ψ can be estimated from unadjusted analyses (i.e., without including any covariates in the regression model) whereas the conditional estimands ν and λ can be estimated from adjusted analyses (i.e., by including all covariates C in the regression model). We will make a distinction, however, and use conditional/marginal to refer to the estimand (as above) and adjusted/unadjusted to refer to the analysis. This is because, as discussed in the next two sections, it is possible to obtain an estimate of the marginal causal log odds ratio ϕ and the marginal causal log hazard ratio κ from analyses that adjust for C (Struthers & Kalbfleisch, 1986; Zhang, 2008). These are examples of adjusted estimators of marginal estimands.

## 3 ESTIMATING THE MARGINAL CAUSAL LOG ODDS RATIO BY REGRESSION ADJUSTMENT

In an RCT there is no need to adjust for C in order to estimate the marginal causal log odds ratio ϕ consistently. Randomization implies that ϕ=β and hence the unadjusted analysis is valid. However, we may wish to adjust for C for a variety of reasons, for example, to increase power, to look at effect modification, and—although not always appreciated—to increase precision.

In an observational study, we are likely to want to adjust for C to attempt to control for confounding (as well as for the reasons listed above). Suppose counterfactual consistency and conditional exchangeability given C (i.e., assumption (4)) hold, and that model (6) is correctly specified. Then, having used our data on X, Y, and C to estimate the parameters of (5) consistently, for example, by maximum likelihood, our assumptions (of counterfactual consistency and conditional exchangeability given C) imply that our estimator of ν is a consistent estimator of ζ also.

If ζ were the estimand of interest, then we would be done. But there are many settings in which we might be more interested in a marginal estimand (e.g., a marginal risk difference, a marginal risk ratio, or a marginal odds ratio) instead. This could be because we want to compare our results with those from a different study in which a different set of covariates was measured, or from which only an estimate of a marginal odds ratio, say, is available, or to combine our results in a meta‐analysis with those from studies that had a different covariate set. Since the magnitude of a non‐null conditional log odds ratio can (if we take the philosophical viewpoint of deterministic potential outcomes) be made arbitrarily large by including more and more covariates in the model (in the extreme, if all causes of the outcome other than the exposure were included in the model, then the true conditional log odds ratio for a non‐null exposure effect would be either positive or negative infinity) then, some would argue, a marginal odds ratio (or a conditional odds ratio conditional on only a subset of the covariate set) is more meaningful.

Whatever the reason might be, if, having fitted a logistic regression model conditional on C, we are still interested in the marginal causal log odds ratio ϕ, it is straightforward to use the output of our analysis to construct an estimator of ϕ, as we now review (Zhang, 2008). Note also that the ‐margins‐ command in both Stata and R perform the steps we outline below.

By the rule of iterated expectations, E(A)=E{E(A|B)}, we have that

(11) $$\begin{array}{cc} \mathrm{Pr}\left(Y_{x}=1\right) & =E\left\{\mathrm{Pr}\left(Y_{x}=1\left|C\right)\right)\right\}\\ & =\int \mathrm{Pr}\left(Y_{x}=1\left|C=c\right)\right)f_{C}\left(c\right)\;dc, \end{array}$$

where $f_{C}\left(c\right)$ is the probability density function for C, which can be replaced by a probability mass function for discrete covariates, or a density with respect to a suitable dominating measure for a mixture of the two.

We have a consistent estimator of $Pr(Y_{x}=1|C=c)$ from our estimators of the parameters of model (5) (since our assumptions allow us to equate these to the parameters of model (6)):

(12) $$\begin{array}{cc} \hat{\mathrm{Pr}}\left(Y_{x}=1\left|C=c\right)\right) & =\mathrm{expit}\left(\hat{\eta }+\hat{\zeta }x+{\hat{\tau }}^{T}c\right)\\ & =\mathrm{expit}\left(\hat{\mu }+\hat{\nu }x+{\hat{\gamma }}^{T}c\right), \end{array}$$

where $expit\left(z\right):=\frac{\exp(z)}{1+\exp(z)}$.

We can plug this into (11) and use the empirical distribution of C as a nonparametric estimator of $f_{C}\left(c\right)$. This leads to the estimator

(13) $$\begin{array}{cc} \hat{\mathrm{Pr}}\left(Y_{x}=1\right) & =\frac{1}{n}\sum_{i=1}^{n}\hat{\mathrm{Pr}}\left(Y_{x}=1\left|C_{i}\right)\right)\\ & =\frac{1}{n}\sum_{i=1}^{n}\mathrm{expit}\left(\hat{\mu }+\hat{\nu }x+{\hat{\gamma }}^{T}C_{i}\right) \end{array}$$

for $Pr(Y_{x}=1)$, where $C_{i}$ are the covariate values observed for individual i in our study (i=1,…,n).

Finally, by evaluating this for x=1 and x=0 and finding the log of the ratio of the two resulting odds, we have our covariate‐adjusted estimator of ϕ:

(14) $$\begin{array}{cc} {\hat{\phi }}^{C-A} & =log\left\{\frac{\hat{Pr}\left(Y_{1}=1\right)}{1-\hat{Pr}\left(Y_{1}=1\right)}\right\}-log\left\{\frac{\hat{Pr}\left(Y_{0}=1\right)}{1-\hat{Pr}\left(Y_{0}=1\right)}\right\}\\ & =log\left\{\frac{\sum_{i=1}^{n}expit\left(\hat{\mu }+\hat{\nu }+{\hat{\gamma }}^{T}C_{i}\right)}{n-\sum_{i=1}^{n}expit\left(\hat{\mu }+\hat{\nu }+{\hat{\gamma }}^{T}C_{i}\right)}\right\}-log\left\{\frac{\sum_{i=1}^{n}expit\left(\hat{\mu }+{\hat{\gamma }}^{T}C_{i}\right)}{n-\sum_{i=1}^{n}expit\left(\hat{\mu }+{\hat{\gamma }}^{T}C_{i}\right)}\right\}. \end{array}$$

We stress that this is a *covariate‐adjusted estimator of the marginal causal log odds ratio*. Whenever γ≠0, ${\hat{\phi }}^{C-A}$ is asymptotically more efficient than the corresponding unadjusted estimator. Thus covariate adjustment is useful even when there is no confounding; in such a situation, the unadjusted estimator is consistent but inefficient.

Let ${\hat{\phi }}^{U}$ be the usual MLE of ϕ from an unadjusted analysis of an RCT, that is, ${\hat{\phi }}^{U}=\hat{\beta }$ where $\hat{\beta }$ is the usual MLE of β. Let AV stand for asymptotic variance, and suppose all models involved are correctly specified and (4) holds. Then, although, as we noted previously,

$$\mathrm{AV}\left(\hat{\nu }\right)\geq \mathrm{AV}\left(\hat{\beta }\right),$$

which implies that,

$$\mathrm{AV}\left(\hat{\zeta }\right)\geq \mathrm{AV}\left({\hat{\phi }}^{U}\right),$$

we have that

$$\mathrm{AV}\left({\hat{\phi }}^{U}\right)\geq \mathrm{AV}\left({\hat{\phi }}^{C-A}\right).$$

In other words, as soon as we compare two apples (as opposed to an orange and an apple), we see that covariate‐adjustment does indeed lead to increased efficiency in logistic regression (Moore & van der laan, 2009).

Again, since ${\hat{\phi }}^{C-A}$ is a covariate‐adjusted estimator of the marginal causal log odds ratio, it illustrates that “conditional” and “adjusted” should *not* be used interchangeably.

Approximate statistical inference about ${\hat{\phi }}^{C-A}$ is possible via the delta method (the preferred option for anyone opposed to the use of random numbers in inference) but the nonparametric bootstrap will usually perform better, be easier to implement, with an acceptable computational cost in many settings.

## 4 PROPOSED PROCEDURE FOR ESTIMATING (BY SIMULATION) MARGINAL CAUSAL HAZARD RATIOS BY REGRESSION ADJUSTMENT

We propose a similar procedure for estimating marginal causal hazard ratios after first fitting a Cox model conditional on covariates C. The first half of the procedure follows exactly the suggestion made by Hernán (2010) and others (see, e.g., the discussion of expected survival in Therneau and Gramsch (2000)) for estimating marginal causal survivor functions following the fitting of a conditional Cox model. We simply append to this a practical suggestion for how to use these survivor functions to estimate a marginal causal hazard ratio. Struthers and Kalbfleisch (1986) give a theoretical result on marginalizing conditional hazard ratios; our proposal is simply an implementation by simulation of this result, which can be used when the analytical solution is intractable.

### 4.1 Estimating the marginal survivor functions under assignment to both exposures

Having fitted model (8), under the assumptions of counterfactual consistency, conditional exchangeability and correct parametric specification of (8), our estimators are consistent estimators of the parameters of model (9):

$${\hat{\tilde{h}}}_{x}^{*}\left(t|c\right)={\hat{\tilde{h}}}_{0}^{*}\left(t\right)\exp\left(\hat{\omega }x+{\hat{\xi }}^{T}c\right)={\hat{\tilde{h}}}_{0}\left(t\right)\exp\left(\hat{\lambda }x+{\hat{\rho }}^{T}c\right),$$

where ${\hat{\tilde{h}}}_{0}\left(t\right)$ is the usual nonparametric estimator of the baseline hazard function as described in section 4.3 of Kalbfleisch and Prentice (2011).

We thus have, using standard relationships between hazard and survivor functions:

$${\hat{S}}_{x}\left(t|c\right):=1-\hat{Pr}\left(T_{x}\leq t|C=c\right)={\left[\exp\left\{-\int_{0}^{t}{\hat{\tilde{h}}}_{0}\left(s\right)ds\right\}\right]}^{\exp\left(\hat{\lambda }x+{\hat{\rho }}^{T}c\right)},$$

which can then be nonparametrically averaged over the distribution of C to give:

(15) $${\hat{S}}_{x}\left(t\right):=1-\hat{Pr}\left(T_{x}\leq t\right)=\frac{1}{n}\sum_{i=1}^{n}{\left[\exp\left\{-\int_{0}^{t}{\hat{\tilde{h}}}_{0}\left(s\right)ds\right\}\right]}^{\exp\left(\hat{\lambda }x+{\hat{\rho }}^{T}C_{i}\right)}.$$

### 4.2 Simulating and analyzing event times under the two marginal survival distributions

We then propose that 2m survival times be simulated (where m≫n: the higher the value of m we choose, the less Monte Carlo error there will be in our estimate of κ), with m survival times simulated according to ${\hat{S}}_{1}\left(\cdot \right)$, and m according to ${\hat{S}}_{0}\left(\cdot \right)$, as described below. When using this method in practice, it would be sensible to increase m until repeated analyses with a different seed give the same results to as many decimal places as the results are quoted.

#### 4.2.1 In the absence of censoring

We first outline the steps under the (usually false) supposition that there is no censoring in the original data, a supposition that we then go on to relax. In the absence of censoring in the original data, the simulation would be performed as follows. The rationale for each step is given below, and so readers may find it helpful to read the rationale first.

1. For j=1,…,m, let $Z_{0,j}^{1}=Z_{0,j}^{0}=1$.
2. Let $t_{1}<\cdots <t_{k}$ be the ordered event times in the original data set, set $t_{0}=0$ and ${\hat{S}}_{1}\left(0\right)={\hat{S}}_{0}\left(0\right)=1$. Then, for x=0,1, for each l=1,…,k and for each j=1,…,m, simulate $Z_{l,j}^{x}$ from a Bernoulli distribution with mean ${\hat{S}}_{x}\left(t_{l}\right)/{\hat{S}}_{x}\left(t_{l-1}\right)$.
  Note that in the absence of censoring, k=n, but later it will matter that $t_{1}<\cdots <t_{k}$ be event times, and that the censoring times of censored individuals should not be included. Note also that were there to be tied event times, these should be artificially perturbed by a very small amount to ensure $t_{1}<\cdots <t_{k}$.
3. For each x=0,1 and j=1,…,m, let
  $${\tilde{D}}_{x,j}=1-\prod_{l=1}^{k}Z_{l,j}^{x}$$
  and
  $${\tilde{Y}}_{x,j}=\left(1-{\tilde{D}}_{x,j}\right)t_{k}+{\tilde{D}}_{x,j}\min\left\{t_{l}:Z_{l,j}^{x}=0\right\}.$$
4. For j=1,…,m, let $\left({\tilde{X}}_{j},{\tilde{D}}_{j},{\tilde{Y}}_{j}\right)=\left(0,{\tilde{D}}_{0,j},{\tilde{Y}}_{0,j}\right)$. For j=m+1,…,2m, let $\left({\tilde{X}}_{j},{\tilde{D}}_{j},{\tilde{Y}}_{j}\right)=\left(1,{\tilde{D}}_{1,j-m},{\tilde{Y}}_{1,j-m}\right)$.
5. Using all records from j=1 to 2m, fit a Cox model with right‐censored event time $\tilde{Y}$, event indicator $\tilde{D}$ (where $\tilde{D}=1$ indicates that $\tilde{Y}$ is an event time and $\tilde{D}=0$ indicates that $\tilde{Y}$ is a censoring time) and the single covariate $\tilde{X}$.
6. The maximum partial likelihood estimator of the coefficient of $\tilde{X}$ in this model is ${\hat{\kappa }}^{C-A}$, our covariate‐adjusted estimator of the marginal causal log hazard ratio κ from model (7).

#### 4.2.2 Rationale for steps 1–6

Steps 1 and 2 use the estimated marginal causal survival curves to simulate time‐to‐event data in a discrete manner. The study period for the original data is divided into discrete windows, defined by the event times in the original data. At time $t_{0}=0$, everyone in the simulated data is still a survivor. By the end of the window $\left(0,t_{1}\right]$, a proportion ${\hat{S}}_{x}\left(t_{1}\right)$ still survives. The conditional probability of surviving the next window, $\left(t_{1},t_{2}\right]$, conditional on surviving the first window, is ${\hat{S}}_{x}\left(t_{2}\right)/{\hat{S}}_{x}\left(t_{1}\right)$, and so on. The simulated binary $Z_{l,j}^{x}$ is thus 0 if and only if simulated individual j experienced the event at the end of window l when assigned to exposure x. The simulated event time for j under exposure x is the time of the end of the first window at which an event is simulated to occur. If an event is never simulated to occur (i.e., $Z_{l,j}^{x}=1$ for all l) then individual j is censored at the final event time $t_{k}$. This is what step 3 achieves. Step 4 prepares the simulated data for the marginal Cox PH analysis by stacking all 2m individuals on top of each other, with their appropriate censored event times and event indicators, and sets the exposure to 0 for the m simulations made under ${\hat{S}}_{0}\left(\cdot \right)$, and to 1 for the m simulations made under ${\hat{S}}_{1}\left(\cdot \right)$. Steps 5 and 6 are then self‐explanatory.

#### 4.2.3 Time frame

It is important to note that, since we assume that model (8) is correctly specified, then the model fitted in step 5 will, in general, be misspecified. That is, under model (8) and our structural assumptions, the true marginal causal hazard ratio will, in general, vary over time in a way that we are ignoring. This is why we should be careful in our choice of time frame over which to simulate data when carrying out our proposed estimation method.

One obvious choice is to select the same time frame as the original study, so that the implicit averaging of the time‐varying hazard ratio happens over the same range of times as in the study. This is especially the case if our original data arose from an RCT, with the covariate‐adjustment procedure a means for gaining efficiency rather than correcting bias. In this case, by selecting the same time frame, our covariate‐adjusted estimator ${\hat{\kappa }}^{C-A}$ described above will have the same mean as (but increased precision compared with) the unadjusted estimator from the usual marginal Cox model fitted to the original data.

Depending on the context in which this method is being used, it could be sensible to choose a shorter time frame than that of the study (but note that the data would not allow a longer time frame to be considered without extending the method to include hard‐to‐justify parametric assumptions for extrapolation). For example, if the aim is to compare (and possibly combine) different HR estimates from many studies, then choosing the shortest time frame among all studies, and marginalizing all estimators over this shortest time frame would ensure as close as possible to a like‐with‐like comparison from this point of view.

Note that the choice of time frame affects not just the implementation of the estimation by simulation, but also the very definition of the marginal estimand, which is as the probability limit (as the sample size →∞) of the marginal hazard ratio that would be estimated from an RCT *of the chosen length*. Often, but not always, the chosen length will be the same as the length of the original study.

#### 4.2.4 In the presence of censoring

For the same reason that we must be careful about the simulated time frame, we must also consider how censoring is simulated. Again, one seemingly obvious choice is to simulate censoring according to the same structure as is seen in the original data. This is especially the case when the aim is simply to use covariates to gain efficiency when estimating a marginal HR in a single RCT. However, in other contexts it could be sensible to simulate under a different censoring distribution, for example “no censoring except at the study end,” or indeed to mimic the censoring distribution seen in a different study with which the results of the study at hand will be compared.

To simulate censoring from the same distribution as is estimated to operate in the study, we propose that (instead of steps 4–6 above) steps 1–3 above be followed by:

4'. In the original data set, let $D^{*}=1-D$ so that being censored is the event of interest and those experiencing the original event are censored.
5'. Fit a conditional Cox model with right‐censored event time Y, event indicator $D^{*}$ and covariates X and C. If $T^{*}$ represents the hypothetical “uncensored” event time in this scenario, that is, the time at which censoring would happen were the event of interest to be avoided for all, then this model is:
  $${\tilde{h}}^{c}ens\left(t|x,c\right):=\lim_{\Delta t\to 0}\frac{Pr\left(t\leq T^{*}<t+\Delta t|X=x,C=c,T^{*}\geq t\right)}{\Delta t}={\tilde{h}}^{c}ens_{0}\left(t\right)\exp\left(\chi x+\iota^{T}c\right).$$
6'. As was done above for the marginal causal survivor function for the event of interest, we now repeat for the marginal causal survivor function for censoring:
  $${\hat{S}}^{c}ens_{x}\left(t\right):=1-\hat{Pr}\left(T_{x}^{*}\leq t\right)=\frac{1}{n}\sum_{i=1}^{n}{\left[\exp\left\{-\int_{0}^{t}{\hat{\tilde{h}}}^{c}ens_{0}\left(s\right)ds\right\}\right]}^{\exp\left(\hat{\chi }x+{\hat{\iota }}^{T}C_{i}\right)}.$$
7'. In the next three steps, we perform steps 1–3 above again, but with censoring and the event of interest interchanged. That is, for j=1,…,m, let $V_{0,j}^{1}=V_{0,j}^{0}=1$.
8'. Let $t^{c}ens_{1}<\cdots <t^{c}ens_{k^{c}}$ be the ordered censoring times in the original data set, set $t^{c}ens_{0}=0$ and ${\hat{S}}^{c}ens_{1}\left(0\right)={\hat{S}}^{c}ens_{0}\left(0\right)=1$. Then, for x=0,1, for each $l=1,\ldots ,k^{c}$ and for each j=1,…,m, simulate $V_{l,j}^{x}$ from a Bernoulli distribution with mean ${\hat{S}}^{c}ens_{x}\left(t_{l}\right)/{\hat{S}}^{c}ens_{x}\left(t_{l-1}\right)$.
9'. For each x=0,1 and j=1,…,m, let
  $${\tilde{D}}_{x,j}^{*}=1-\prod_{l=1}^{k^{c}}V_{l,j}^{x}$$
  and
  $${\tilde{Y}}^{c}ens_{x,j}=\left(1-{\tilde{D}}_{x,j}^{*}\right)t_{k^{c}}+{\tilde{D}}_{x,j}^{*}\min\left\{t_{l}:V_{l,j}^{x}=0\right\}.$$
10'. Now, step 4 above is replaced with the following. For j=1,…,m, let
  $$\left({\tilde{X}}_{j},{\tilde{D}}_{j},{\tilde{Y}}_{j}\right)=\left(0,I\left({\tilde{Y}}_{0,j}<{\tilde{Y}}_{0,j}^{cens}\right)\cdot {\tilde{D}}_{0,j},\min\left\{{\tilde{Y}}_{0,j},{\tilde{Y}}_{0,j}^{cens}\right\}\right).$$
  For j=m+1,…,2m, let
  $$\left({\tilde{X}}_{j},{\tilde{D}}_{j},{\tilde{Y}}_{j}\right)=\left(1,I\left({\tilde{Y}}_{1,j-m}<{\tilde{Y}}_{1,j-m}^{cens}\right)\cdot {\tilde{D}}_{1,j-m},\min\left\{{\tilde{Y}}_{1,j-m},{\tilde{Y}}_{1,j-m}^{cens}\right\}\right).$$

Finally, we perform steps 5 and 6, exactly as described in Section 4.2.1.

#### 4.2.5 Rationale for steps 4'–10'

Steps 4'–9' mimic what was done up to step 3 but with the event of interest and censoring interchanged, so that censoring can be simulated in a way that mimics the original data. This interchanging of event and censoring times is justified by the independent censoring assumption. Step 10' simply compares the simulated event and censoring times for each individual and chooses the earlier of the two as the censored event time, generating the appropriate event indicator to reflect this. The only slight additional complication is that for some individuals, even their simulated “event time” was in fact a censoring time, and this is reflected in the way in which the event indicator is defined.

### 4.3 Statistical inference

If we consider step –1 to be the fitting of (8), and step 0 to be the prediction of the marginal causal survivor functions given in (15), then the entire estimation procedure consists of following 14 steps, namely, –1, 0, 1–3, 4'–10', 5, 6, in that order. For inference, we propose that all 14 steps be performed within a nonparametric bootstrap.

## 5 SIMULATION STUDY

In describing the design and results of our simulation study, we follow the guidelines set out by Morris, White, and Crowther (2019). R code is included in the Supplementary Material.

### 5.1 Design

#### 5.1.1 Aims

Our main aim is to verify that our proposal for approximating the covariate‐adjusted estimator of the marginal hazard ratio by Monte Carlo simulation performs well in terms of bias and precision in both an RCT and an observational study setting. Our secondary aims—and for these we include settings with a binary outcome as well as a time‐to‐event outcome—are (a) to illustrate empirically some of the theoretical comparative properties (attenuation, precision) of the various estimators discussed earlier in the paper, and (b) to investigate the Monte Carlo error due to different choices of m in our proposal.

#### 5.1.2 Data generating mechanisms

There are eight data generating mechanisms in total: four with a binary outcome and four with a time‐to‐event outcome. Within each set of four, three are for an RCT setting, and the fourth is an observational study. Within each set of three RCT settings, the first has a null exposure (treatment) effect on the outcome and a non‐null effect of a baseline covariate on the outcome, the second has a non‐null exposure effect and no covariate effect, and the third has both a non‐null exposure and a non‐null covariate effect. In the observational study setting, there is both a non‐null exposure effect and a non‐null covariate effect, and furthermore this covariate is a confounder since it also has a non‐null effect on the exposure. We now give more details on the precise generating distributions for each scenario.

All eight scenarios start by simulating a single covariate C once from a normal distribution with mean 0 and variance 1, and these values remain fixed, within individual, across simulations.

The exposure X is resimulated in each new simulated data set to follow a Bernoulli distribution with probability either 0.5 or expit(C) depending on whether or not we are in one of the six RCT scenarios or one of the two observational study scenarios, respectively.

In the three RCT scenarios with a binary outcome, the outcome Y is resimulated in each new simulated data set to follow a Bernoulli distribution as given in (5) with μ=1 and (ν,γ) set to (0,1), (1,0), or (1,1), respectively, in accordance with the descriptions given above. In the observational study scenario with a binary outcome, Y is simulated in exactly the same way as for the third RCT scenario, that is, with (ν,γ)=(1,1). As a short hand, we will refer to the three RCT scenarios as (0,1), (1,0), and (1,1), and the observational study scenario as ${\left(1,1\right)}^{*}$.

In the four scenarios with a time‐to‐event outcome, individuals are simulated to enter the study uniformly over 2 years, and their event time is then simulated to occur at a random time T years after this entry time, where T|X,C is simulated from a Weibull distribution with scale parameter 0.1 and shape parameter 1.5 and linear predictor parameters (corresponding to λ and ρ in (8)) of (0,1), (1,0), and (1,1), respectively. All individuals who have not yet experienced the event are administratively censored at 10 years since the start of the recruitment window, and the time‐scale for analysis is time since recruitment. This therefore corresponds to a PH distribution conditional on C, with independent right‐censoring occurring during follow‐up. Again, we repeat the third scenario for an observational study (with Pr(X=1|C)=expit(C) instead of Pr(X=1|C)=0.5), and denote this scenario as ${\left(1,1\right)}^{*}$.

#### 5.1.3 Estimands of interest

There are six estimands of interest, with the first three relevant to the scenarios with a binary outcome, and the remaining three relevant to the scenarios with a time‐to‐event outcome:

1. β: the marginal associational log odds ratio
2. ϕ: the marginal causal log odds ratio
3. ν=ζ: the conditional log odds ratio, conditional on C (this has both a causal and associational interpretation)
4. ψ: the marginal associational log hazard ratio
5. κ: the marginal causal log hazard ratio
6. λ=ω: the conditional log hazard ratio, conditional on C (this has both a causal and associational interpretation).

#### 5.1.4 Methods to be compared

The following methods are compared:

(A) an unadjusted logistic or Cox model,
(B) the same unadjusted model as in (A) but including inverse probability of treatment weighting (IPTW) (Hernan, 2006), where the model for the treatment weights is a logistic regression for treatment/exposure given the baseline covariate/confounder,
(C) Zhang's method (for binary outcomes) or our proposal (for right‐censored time‐to‐event outcomes),
(D) an adjusted logistic or Cox model including the baseline covariate/confounder where we are simply interested in the estimator of the conditional log OR or log HR from these models.

Note that (A)–(C) deliver estimates of the marginal log OR or log HR, whereas (D) delivers an estimate of the conditional log OR or log HR given C. When simulating data from an RCT, all four analyses deliver estimators of causal estimands, whereas this is only the case in the observational setting for analyses (B)–(D). That is, in the three RCT scenarios, (A)–(C) deliver estimates of β=ϕ (binary outcome) or ψ=κ (time‐to‐event outcome). In the observational study scenario, (A) delivers an estimate of β or ψ, whereas (B) and (C) deliver estimates of ϕ or κ. In all scenarios, (D) delivers an estimate of ν=ζ (binary outcome) or λ=ω (time‐to‐event outcome).

#### 5.1.5 Sample size, number of simulations, and performance measures

Each simulated data set contains 1000 individuals. This sample size is chosen since finite sample (or sparse data) bias (Greenland, Manrournia, & Altman, 2016) is then negligible for the data generating mechanisms we use, and since sparse data bias, although important in logistic and Cox PH regression, is not the focus of this article.

From each of the eight data generating mechanisms, we generate 1000 data sets. This number was chosen by trial and error so that our simulation study aims could be addressed with sufficient confidence that our conclusions were not due to chance, where “chance” here refers to not having simulated a sufficient number of data sets. To illustrate, in scenario (1,1) (non‐null treatment and covariate effect) with a time‐to‐event outcome, our proposed estimator of the marginal log HR appears to be more efficient ($\hat{SE}=0.0499$) than the unadjusted estimator ($\hat{SE}=0.0663$). These two estimated SEs themselves are estimated with SE 0.0011 and 0.0015, respectively (Morris et al., 2019). Thus, even if we ignore the correlation between the two estimated SEs (which we expect to be >0 given that the *same* 1,000 simulated data sets are used in both), the Z‐score for the difference between the two estimated SEs is 8.8, rendering Monte Carlo error (due to an insufficient number of simulated data sets in the simulation study) a highly unlikely explanation for this difference in efficiency; likewise, the other observations made. In the light of the magnitude of the simulation Monte Carlo SEs estimated (using the formulae given in Morris et al. (2019)), and included in our table of simulation study results (see Table 1), we henceforth quote our results to 2 two decimal places.

**TABLE 1:** Results of the simulation study

			Scenarios				
	Methods		Performance measure	Null treatment effect, Covariate effect (0,1)	Treatment effect, Null covariate effect (1,0)	Treatment effect, Covariate effect (1,1)	Treatment effect, Confounder effect ${\left(1,1\right)}^{*}$
Binary outcome	(A) Unadjusted		Mean (MC error${}^{1}$)	−0.01 (0.0045)	1.00 (0.0055)	0.86 (0.0051)	1.60 (0.0054)
			Empirical SE (MC error)	0.14 (0.0032)	0.17 (0.0039)	0.16 (0.0036)	0.17 (0.0038)
	(B) IPTW		Mean (MC error)	−0.00 (0.0042)	1.00 (0.0055)	0.87 (0.0048)	0.87 (0.0059)
			Empirical SE (MC error)	0.13 (0.0030)	0.17 (0.0039)	0.15 (0.0034)	0.19 (0.0042)
	(C) Adjusted marginal		Mean (MC error)	−0.00 (0.0042)	1.00 (0.0055)	0.87 (0.0048)	0.87 (0.0054)
	(Zhang's method)		Empirical SE (MC error)	0.13 (0.0029)	0.17 (0.0039)	0.15 (0.0034)	0.17 (0.0038)
	(D) Conditional		Mean (MC error)	−0.00 (0.0049)	1.00 (0.0055)	1.00 (0.0055)	1.00 (0.0059)
			Empirical SE (MC error)	0.15 (0.0035)	0.17 (0.0039)	0.17 (0.0039)	0.19 (0.0042)
Time‐to‐event	(A) Unadjusted		Mean (MC error)	−0.00 (0.0021)	1.00 (0.0021)	0.66 (0.0021)	1.26 (0.0023)
outcome			Empirical SE (MC error)	0.07 (0.0015)	0.07 (0.0015)	0.07 (0.0015)	0.07 (0.0016)
	(B) IPTW		Mean (MC error)	−0.00 (0.0017)	1.00 (0.0021)	0.66 (0.0017)	0.67 (0.0025)
			Empirical SE (MC error)	0.05 (0.0012)	0.07 (0.0015)	0.05 (0.0012)	0.08 (0.0017)
	(C) Adjusted marginal	2m=1000	Mean (MC error)	−0.00 (0.0026)	1.00 (0.0032)	0.66 (0.0026)	0.66 (0.0029)
	(our proposal)		Empirical SE (MC error)	0.08 (0.0018)	0.10 (0.0022)	0.08 (0.0018)	0.09 (0.0020)
		2m=5000	Mean (MC error)	−0.00 (0.0017)	1.00 (0.0024)	0.66 (0.0017)	0.66 (0.0020)
			Empirical SE (MC error)	0.05 (0.0012)	0.08 (0.0017)	0.05 (0.0012)	0.06 (0.0014)
		2m=10000	Mean (MC error)	−0.00 (0.0016)	1.00 (0.0023)	0.66 (0.0016)	0.66 (0.0018)
			Empirical SE (MC error)	0.05 (0.0011)	0.07 (0.0016)	0.05 (0.0011)	0.06 (0.0013)
	(D) Conditional		Mean (MC error)	−0.00 (0.0021)	1.00 (0.0022)	1.00 (0.0022)	1.00 (0.0024)
			Empirical SE (MC error)	0.07 (0.0015)	0.07 (0.0015)	0.07 (0.0016)	0.08 (0.0017)

*Note*. The “MC error” here refers to the Monte Carlo standard errors of our estimators of the two performance measures, as estimated from 1000 simulated data sets, and are calculated using the formulæ given in Morris et al. (2019). These MC errors would decrease had we chosen a larger number of simulated data sets for our simulation study.

For each method under each scenario, we obtain 1000 estimates of the relevant estimand and then we calculate the sample mean and standard deviation of these 1000, which respectively are our simulation estimators of the mean and empirical standard error of each estimator (the performance measures in our simulation study).

### 5.2 Results

#### 5.2.1 Binary outcome

**FIGURE 4:**
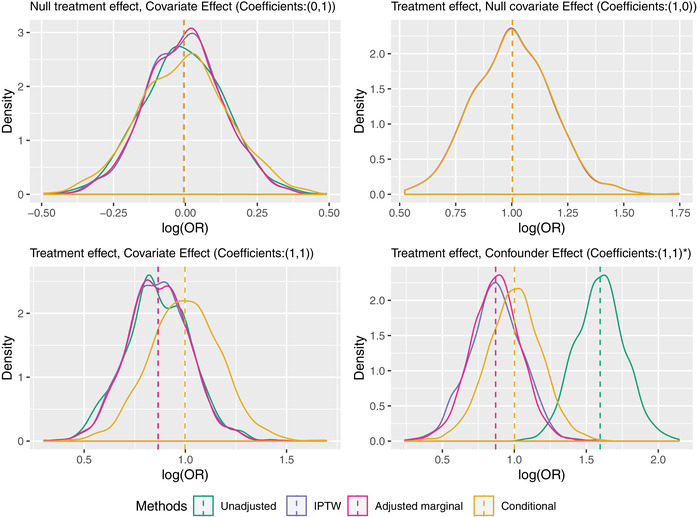
Kernel density plots of the estimates from the 1000 simulated data sets with a binary outcome

The results for the four different estimators in the four scenarios are given in the top half of Table 1 and further illustrated by the kernel density plots in Figure 4.

These simulation results illustrate several aspects of the established theoretical results discussed in earlier sections. First, by looking at the mean of the estimates, we see that noncollapsibility is present if and only if there is both a non‐null association between X and Y conditional on C and between C and Y conditional on X (therefore not in scenarios (0,1) or (1,0)). In scenario (1,1), noncollapsibility is demonstrated and in the direction predicted, with the marginal log odds ratio closer to the null than the conditional log odds ratio, in a setting with no confounding. In scenario ${\left(1,1\right)}^{*}$, both noncollapsibility and confounding are present, and, in this particular set‐up, they “pull” the unadjusted odds ratio in opposite directions. The fact that the mean of the estimates for both IPTW and Zhang's proposal are very similar in scenarios (1,1) and ${\left(1,1\right)}^{*}$ demonstrates that both methods are successfully reweighting and adjusting for the confounding, respectively, and consistently estimating the marginal causal log odds ratio from the observational study.

As for efficiency, we see that, as theory dictates, and as is often quoted, the standard error of the estimator of the conditional log odds ratio (method D) is at least as large as the standard error of the marginal log odds ratio from the unadjusted analysis (A), from which we might conclude that covariate adjustment (while useful for bias in scenario ${\left(1,1\right)}^{*}$) is otherwise detrimental to precision and hence not desirable in RCTs. However, by ‘making an apple out of the orange’ (Zhang's method) and hence comparing estimators of the same marginal causal log odds ratio (in the first three scenarios), we find that covariate‐adjustment leads to a gain in efficiency whenever the covariate is predictive of the outcome (i.e., in the first and third scenarios). Interestingly, this gain in efficiency looks identical to that achieved by IPTW in these scenarios, suggesting that the result proved by Williamson, Forbes, and White (2014) for linear regression holds more generally. In an observational study, however, covariate adjustment is in general *more* efficient than IPTW, and this is demonstrated by the relative standard errors of Zhang's method and IPTW in the final scenario.

#### 5.2.2 Right‐censored time‐to‐event outcome

**FIGURE 5:**
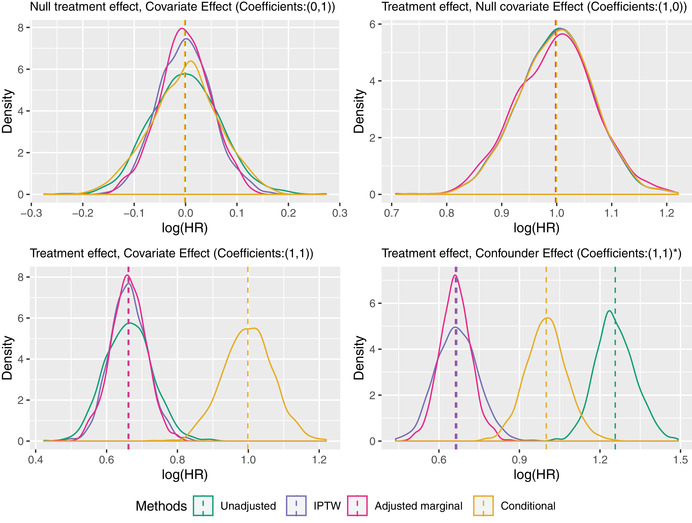
Kernel density plots of the estimates from the 1000 simulated data sets with a time‐to‐event outcome. Note that 2m=10000 in the variant of our proposal (“adjusted marginal”) presented here

The results for the four different methods in the four scenarios are given in the bottom half of Table 1 and further illustrated by the kernel density plots in Figure 5.

The observations made for the simulation results with a binary outcome can almost be repeated verbatim here. One important caveat, however, is that the superior efficiency of our proposal relative to IPTW (in scenario ${\left(1,1\right)}^{*}$) and to the unadjusted analysis (in scenarios (0,1) and (1,1)) is only evident when m is sufficiently large. For smaller values of m, the superior efficiency of covariate adjustment is more than counterbalanced by the Monte Carlo error introduced by the simulations involved in our proposal (due to not choosing an infinite value of m). When using our proposal in practice, it will therefore always be advisable to repeat the analysis with increasing values of m until no appreciable further changes either in the point estimate or the bootstrap estimate of SE are seen.

**FIGURE 6:**
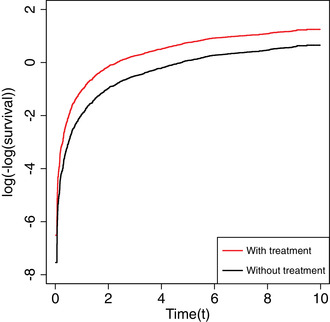
Complementary log‐log survival against time plotted for each of the two marginal survivor functions in (15) as estimated for the two treatment groups in an example simulated data set

Figure 6 shows the complementary log‐log marginal survival curves $log[-log\left\{{\hat{S}}_{x}\left(t\right)\right\}]$ (see Equation (15)) against time for the two treatment groups separately (x=0,1) as estimated from one example simulated data set from the RCT scenario with both a non‐null treatment and covariate effect (1,1). Recall that we do not assume that the PH assumption holds marginally, and thus we would not typically expect the curves in Figure 6 to be parallel. However, assessing how far from being parallel they appear is relevant for the interpretation of the marginal causal log hazard ratio, which can be thought of as a form of weighted average of the time‐varying log hazard ratio, where the weights reflect the censoring distribution. The interpretation is arguably more straightforward if the two curves are close to being parallel, as indeed they are in this case for the majority of the 10‐year follow‐up.

## 6 APPLICATION TO THE PBC DATA

We applied our proposal in a reanalysis of data analyzed by Therneau and Gramsch (2000) from patients with PBC, a chronic liver disease. In a double‐blind trial conducted at the Mayo Clinic between 1988 and 1992, 188 patients were randomized to receive either a new treatment, ursodeoxycholic acid (UDCA), or placebo, with 95 receiving UDCA and the remaining 93 placebo. The patients were followed up until they experienced one of the following events: death, transplant, histologic progression, development of varices, development of ascites, development of encephalopathy, doubling of bilirubin, or worsening of symptoms. Although this data set has been used to illustrate methods for analyzing times to competing events, our analysis will simply focus on the time to the composite endpoint, that is, the time to whichever of the above events is experienced first. Ninety‐eight patients did not experience any of the events and thus were censored. The data set contains one baseline covariate, namely, log bilirubin at baseline.

We compare the same four methods as used in our simulation study, and again investigate the extent to which our proposal is affected by Monte Carlo error by varying the value of m, and also the time taken to implement our proposal (on a standard desktop PC) as m increases.

The results are given in Table 2. Once again we find largely what we expect, which is that the estimate of the conditional log HR is further away from the null, and has a larger bootstrap SE, than the unadjusted estimate of the marginal log HR, but that this apparent loss of efficiency goes away as soon as we compare like with like, whether this be by comparing IPTW or our proposal to the unadjusted analysis. As noted in our simulation study, for insufficiently large m, the Monte Carlo error in our procedure dominates, but at m≥1,000 this ceases to be the case, and the bootstrap SE for our method is smaller than for the unadjusted estimator, demonstrating the benefit of covariate adjustment for efficiency. The running time for our proposal increases linearly with m.

**TABLE 2:** Results of all 4 methods applied to PBC UDCA data set. All SEs and CIs are based on the nonparametric bootstrap

Method		Estimate	Bootstrap SE	95% CI	Time (mins)
(A) Unadjusted		−0.7503	0.2181	[−1.1852,−0.3347]	0.05
(B) IPTW		−0.7290	0.2159	[−1.1862,−0.3232]	0.18
(C) Adjusted marginal	2m=200	−0.5706	0.2888	[−1.4054,−0.2200]	0.83
(our proposal)	2m=2000	−0.8192	0.2018	[−1.2246,−0.4269]	2.18
	2m=4000	−0.8874	0.1973	[−1.1994,−0.4122]	3.80
	2m=10,000	−0.7631	0.1994	[−1.1817,−0.3976]	7.81
	2m=20,000	−0.7376	0.1991	[−1.1918,−0.3871]	15.46
	2m=40,000	−0.7787	0.2004	[−1.1980,−0.3929]	28.75
	2m=100,000	−0.7914	0.1956	[−1.1758,−0.4005]	72.18
(D) Conditional		−0.8643	0.2253	[−1.3305,−0.4574]	0.05

*Notes*. 2m is the sample size for the simulated data set (m for each exposure level) used within the proposal—see Section 4.2). For all methods, the estimated SE was obtained using the nonparametric bootstrap with 1000 bootstrap samples, and the 95% CI obtained from the 2.5th and 97.5th percentile of the distribution of nonparametric bootstrap estimates.

**FIGURE 7:**
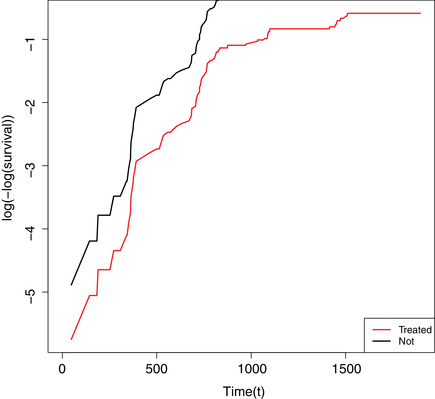
Complementary log‐log survival against time plotted for each of the two marginal survivor functions in (15) as estimated for the two treatment groups in the PBC UDCA data set

Figure 7 shows the complementary log‐log marginal survival curves $log[-log\left\{{\hat{S}}_{x}\left(t\right)\right\}]$ (see Equation (15)) against time for the two treatment groups separately (x=0,1). As discussed in Section 5, the fact that the curves are quite close to being parallel arguably helps to make the interpretation of the marginal causal hazard ratio more straightforward.

## 7 DISCUSSION

In this paper, we have revisited the much‐discussed issue of the noncollapsibility of common effect measures, focussing mainly on odds and hazard ratios in logistic and Cox models. Our initial discussion in Section 1.1, as well as Appendix A.1, include a wider range of link functions and outcome types, via the more general notion of the CCF. This explains why noncollapsibility is not an issue in linear regression models for continuous outcomes, Poisson models for count data, or any other GLM with identity or log link. However, care is needed for models that are not in the GLM family, such as models for rates and hazards, where in general noncollapsibility occurs for all link functions. Additive hazards models are an exception, where the hazard difference is collapsible, as explained in Appendix A.2.

We have described the simple procedure suggested by Zhang for marginalizing estimates of conditional odds ratios after fitting a logistic regression with covariates, and suggested a similar procedure for marginalizing estimates of conditional hazard ratios after fitting a Cox PH regression model with covariates. The procedure for odds ratios is straightforward. Our proposal is slightly more complicated, since care must be taken over time frames and right censoring, but it adheres to the same straightforward principle. It seems surprising, therefore, that this issue is so widely misunderstood, with widespread inappropriate comparisons made of the SEs of estimators of effects conditioned on different covariate sets, as if this provides a meaningful basis for estimator/method choice.

By setting things out as we did in Section 2, the distinction between marginal/conditional estimand and unadjusted/adjusted analysis is easy to make. It seems plausible that the murkiness with which this issue is often described stems from the absence of a mathematical notion of causation in standard statistical notation. This leads to a blurring between estimand and analysis method in traditional statistics, with the analysis method (e.g., covariate‐adjusted or unadjusted) dictating which treatment effect estimand (conditional or marginal, respectively) is implicitly the focus. When viewed through a traditional lens, it is not surprising that ‘conditional’ and ‘adjusted’ became synonymous. Similarly, in observational studies, it is not surprising that marginal effect measures are viewed skeptically, since they are erroneously thought to be tied to unadjusted analyses and hence confounded. In contrast, as soon as formal causal notation is used to distinguish between associational and causal marginal estimands, it is straightforward to see how results from covariate‐adjusted (and hence unconfounded under the usual assumptions) analysis methods can be used to deliver estimators of marginal causal estimands.

Williamson et al. (2014) show that in RCTs with a linear regression model for a continuous outcome, IPTW is asymptotically as efficient as covariate‐adjustment. It is suggested by our simulation results that the same may hold more generally but further work is needed on this. However, in observational studies with any outcome type, covariate adjustment is more efficient than IPTW, as our simulation studies confirm. Arguably, whereas there is more to gain from covariate adjustment as opposed to IPTW in observational studies in terms of efficiency, one needs to balance this against the bias that can be incurred when either the outcome or exposure model is misspecified, an issue we have avoided in this paper. Much literature exists on the robustness or otherwise of various approaches to covariate and confounder adjustment in RCTs and observational studies when parametric nuisance models are misspecified, together with so‐called *double robust* alternatives (Bang & Robins, 2005; Daniel, 2018; Robins, Rotnitzky, & Zhao, 1994; Scharfstein, Rotnitzky, & Robins, 1999; Tsiatis, Davidian, Zhang, & Lu, 2008; Yang & Tsiatis, 2001; Zhang, Tsiatis, & Davidian, 2008).

Although we have focused on the conventional Cox model for covariate adjustment, the marginalization method we propose is equally applicable no matter how we came to our estimate of the conditional survivor function given X and C; for example, we could have used machine learning algorithms. However, the bootstrap estimator of variance would then in general not be valid, and the use of our proposal in conjunction with IPTW in a double robust approach would be preferable (Daniel, 2018). Another setting in which our proposal could be useful is after propensity score adjustment within a Cox model.

Although the proposal as we have described it in detail describes how the simulations should be done so as to mimic the time frame and censoring pattern seen in the data being analyzed, we have also noted that it would be possible to simulate data with a different censoring pattern and/or a shorter time frame. Such a strategy might be desirable when seeking to compare (or combine) results across different studies with different time frames and/or censoring patterns. Note that extending the time frame for simulations would not be possible (except by extrapolation beyond the data using hard‐to‐defend parametric assumptions), however, since the survivor functions are only estimated for the duration of the study being analyzed.

We have supposed throughout that the distribution of covariates over which we wish to marginalize is precisely that seen in the study being analyzed, but there are many situations in which this supposition may be false. For example, if our study recruited twice as many females than males, then it could be desirable (especially if comparing with the marginal estimate from a different study with a 50:50 balance of females and males) to marginalize the estimated conditional log OR or HR over a *balanced* gender distribution. This can relatively easily be done, for example, by including appropriate weights in the sums in (13) and (15).

The previous point is relevant to one of the strongest objections raised to marginal estimands. As Hauck et al. (1998) say, “There is no unique population‐averaged [marginal] treatment effect. Every choice of a set of covariates, including none, is a different population‐averaged model (averaging over all omitted covariates).” Let us consider this remark in the context of an RCT, so that confounding is not relevant. The remark is often used as an argument to prefer conditional estimands, conditional on as many prognostic covariates as possible, so that the set of ‘omitted covariates’ in this quotation is as small as possible. Another popular way of making the same point is to say that conditional effects are more *transportable* (from one population to another) than marginal effects, and are thus of greater scientific relevance. However, as noted in our previous paragraph, any *measured* covariate can be adjusted for in the analysis, and then marginalized over according to any desired reference distribution, resulting in a marginal estimand that is just as transportable as any conditional estimand.

### OPEN RESEARCH BADGES

This article has earned an Open Data badge for making publicly available the digitally‐shareable data necessary to reproduce the reported results. The data is available in the Supporting Information section.

This article has earned an open data badge “**Reproducible Research**” for making publicly available the code necessary to reproduce the reported results. The results reported in this article could fully be reproduced.

## CONFLICT OF INTEREST

The authors have declared no conflict of interest.

## Supporting information

Supporting Information

## Figures and Tables

**FIGURE A.1:**
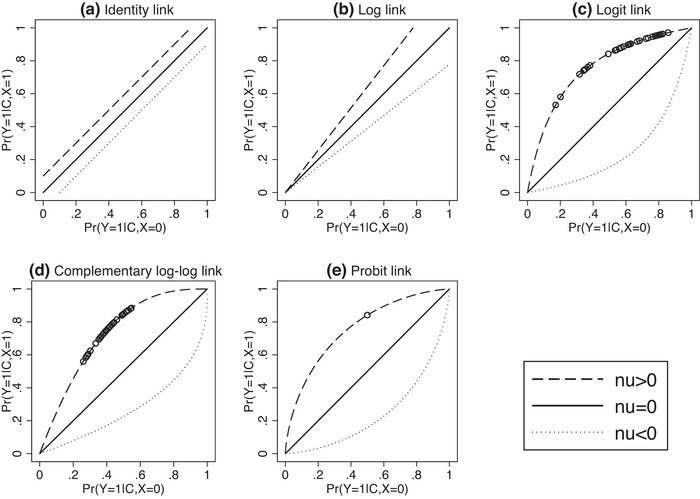
The (non)collapsibility of common effect measures for binary outcomes as determined by the concavity, convexity or linearity of the function $g_{\nu }\left(\cdot \right)=f^{-1}\left\{f\left(\cdot \right)+\nu \right\}$ where f is the link function and ν is the conditional effect measure. f(p)=log{p/(1−p)} for the logit link, f(p)=log(−log(1−p)) for the complementary log‐log link, and $f\left(p\right)=\Phi^{-1}\left(p\right)$, where Φ(·) is the CDF of the standard normal distribution, for the probit link

**FIGURE A.2:**
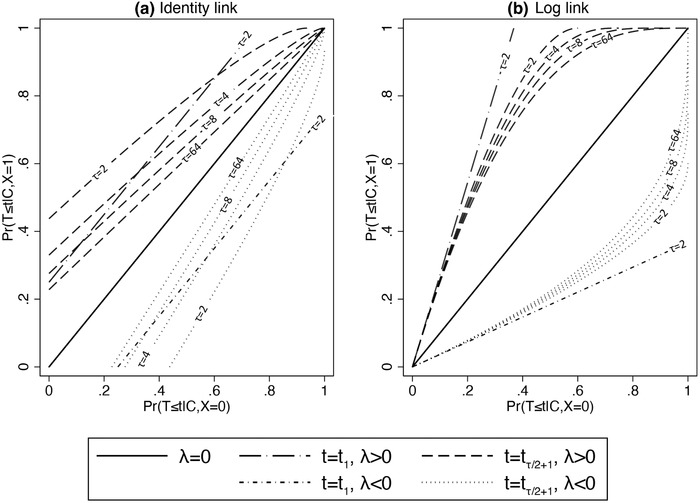
The CCF implied by discrete‐time rate models with (a) an identity link and (b) a log link, for both the first time‐interval, and a subsequent time‐interval, as well as for different values of τ, the total number of time intervals, and for different treatment effect values (λ) on the scale of the linear predictor. As τ→∞, the discrete‐time rate model becomes a continuous‐time hazard model

## Data Availability

The data analyzed in Section 6 are publicly available and can be downloaded from within Stata using the command use http://www.stata-press.com/data/r13/udca.

## APPENDIX A

In the first two appendices, we discuss (non)collapsibility in models for binary and time‐to‐event outcomes in RCTs. Following the logic in Section 2, however, everything that follows applies equally to comparisons between conditional and marginal causal parameters in observational studies. These two appendices contain a more detailed account of the material summarized in Section 1.1 of the main article.

### A.1 Noncollapsibility in models for binary outcomes

Consider models for a binary outcome in an RCT. We consider marginal models:

$$f\left\{Pr(Y=1|X=x)\right\}=\alpha +\beta x$$

and conditional models (conditional on baseline covariates C):

$$f\left\{Pr(Y=1|X=x,C)\right\}=\mu \left(C\right)+\nu x,$$

where f(·) is a link function (such as identity, log, logit), X is the binary treatment and μ(C) is some function of the baseline covariates C, which are independent of X. These are the same as the associational models described in Section 2.1, except that by replacing $\mu +\gamma^{T}C$ with μ(C) (without consequence), and by allowing any link function f, our discussion here is slightly more general.

Writing $p_{x}\left(C\right)$ as shorthand for Pr(Y=1|X=x,C), it is straightforward to show that $p_{1}\left(C\right)$ and $p_{0}\left(C\right)$ are related via a function $g_{\nu }$, say, where

$$g_{\nu }\left(\cdot \right)=f^{-1}\left\{f(\cdot )+\nu \right\}$$

and

$$p_{1}\left(C\right)=g_{\nu }\left\{p_{0}\left(C\right)\right\}.$$

In other words, the conditional probability of Y=1 given C and X=1 is related to the corresponding conditional probability (given C) when X=0 via the function $g_{\nu }$, which first applies the link function—to convert the probability for X=0 to the scale of the linear predictor—then adds the conditional treatment effect (ν) on the scale of the linear predictor, and finally applies the inverse of the link function, returning to the probability scale (now when X=1). As shown by Neuhaus and Jewell (1993), the collapsibility or otherwise of effect measures is inherently linked to this change (and reverse‐change) of scale, and is determined by the nature of $g_{\nu }$, as we now review. We call $g_{\nu }$ the CCF.

Using $p_{x}$ as shorthand for Pr(Y=1|X=x), we have, by the independence of X and C, $p_{1}=E\left\{p_{1}\left(C\right)\right\}=E\left[g_{\nu }\left\{p_{0}\left(C\right)\right\}\right]$. Jensen's inequality, which describes what happens when the order of $g_{\nu }$ and E is reversed is then invoked: if $g_{\nu }$ is concave, $p_{1}\leq g_{\nu }\left[E\left\{p_{0}\left(C\right)\right\}\right]=g_{\nu }\left(p_{0}\right)$, if $g_{\nu }$ is convex, $p_{1}\geq g_{\nu }\left(p_{0}\right)$, with equality if $g_{\nu }$ is linear.

For the identity and log link functions [f(p)=p, f(p)=log(p), respectively], $g_{\nu }\left(p\right)$ is linear in p. For all other common link functions, such as logit [f(p)=log{p/(1−p)}], probit [$f\left(p\right)=\Phi^{-1}\left(p\right),\;\Phi \left(\cdot \right)\;standardnormalCDF$] and complementary log‐log [f(p)=log{−log(1−p)}], $g_{\nu }$ is concave if ν>0 and convex if ν<0, as illustrated in Figure A.1; this can easily be checked by showing that $g_{\nu }^{\prime \prime }\left(\cdot \right)$ is negative (over the domain [0,1]) when ν>0 and positive when ν<0.

Finally, note that $\beta =f\left(p_{1}\right)-f\left(p_{0}\right)$ and $f\left\{g_{\nu }\left(p_{0}\right)\right\}-f\left(p_{0}\right)=\nu$. Thus, if f is an increasing function, as is the case with all common link functions, then depending on whether $g_{\nu }$ is concave or convex, β is either less than or greater than ν, respectively, with equality if $g_{\nu }$ is linear. The above confirms that the marginal parameter β is always at least as close to the null as ν, or |β|≤|ν|: this is why it is often said that noncollapsibility leads to marginal effects that are “attenuated” relative to conditional effects.

When f is the identity link, ν and β are the conditional and marginal risk differences, respectively, and are equal. When f is the log link, ν and β are the conditional and marginal log risk ratios, respectively, and are equal. When f is the logit link, ν and β are the conditional and marginal log odds ratios, respectively, and are *not* in general equal. This is why it is often said that risk differences and ratios are collapsible effect measures, but the odds ratio is not.

Figure A.1 demonstrates why noncollapsibility is a problem for most models for binary data. It is generally seen as a desirable feature that the curves in Figure A.1(c)–A.1(e) coincide at 0 and 1. This is what prevents such models from predicting probabilities outside the range [0,1]. The models depicted by Figure A.1(a) and A.1(b), while they may represent reasonable approximations for some values of $p_{0}\left(C\right)$ and $p_{1}\left(C\right)$, are clearly unrealistic for probabilities close to 1 (in the case of the log link) or close to either 0 or 1 (in the case of the identity link). Noncollapsibility (via the nonlinearity of $g_{\nu }$) is an inevitable consequence of the “bending” of the function that must take place in order to respect the [0,1] boundaries of probabilities.

Figure A.1 also demonstrates the two important exceptions even for the link functions that generally give rise to noncollapsibility. First, when there is no treatment effect (ν=0), $g_{\nu }$ is the identity function irrespective of f, and thus all effect measures are collapsible at the null (which is why null hypothesis significance testing is not affected by noncollapsibility). Second, as the strength of the conditional association between C and Y given X decreases, the relevant points on the graphs in Figure A.1 cluster closer together, and the relevant extent of nonlinearity decreases. In the limit, if there is no association between C and Y conditional on X, there is only one relevant point, the expectation step (over C) can be removed, and all measures are collapsible. We have illustrated decreasing strengths of conditional association between C and Y given X in Figure A.1, with a strong association in (c), a weaker association in (d), and no association in (e).

### A.2 Noncollapsibility in models for time‐to‐event outcomes

In models for time‐to‐event outcomes, the probability (risk) above is replaced by a rate or hazard. In contrast to binary outcomes (where the logit link, via logistic regression, is the usual choice), the most commonly used link functions for time‐to‐event models are the log link (e.g., in the Cox PH model) and the identity link (e.g., in the Aalen additive hazards model). It might be tempting to think, therefore, that noncollapsibility is not an issue for rate/hazard differences/ratios. Sjölander et al. (2015) explain why this reasoning is faulty, and the fact that rates are based on conditional probabilities (conditional on survival) turns out to be crucial. They show that the rate difference and rate ratio are both noncollapsible, as is the hazard ratio, but that (via a slightly more complicated argument than that given in Section A.1. above), the hazard difference is collapsible. We review the arguments by Sjölander et al. (2015) in this section, relating them more closely to the work by Neuhaus and Jewell (1993) described above.

A time‐to‐event outcome T defined in discrete time (such that $T\in \{t_{1},t_{2},\ldots \}$) can be described using a sequence of binary variables $Y_{1},Y_{2},\ldots$, where

$$Y_{s}=\left\{\begin{array}{c} 1\;\mathrm{if}\;T\leq t_{s}\\ 0\;\mathrm{if}\;T\geq t_{s+1} \end{array}\right)$$

Marginal rate models, with a suitable link function f(·), are defined as:

(A.16) $$f\left\{\frac{Pr(Y_{s}=1|Y_{s-1}=0,X=x)}{t_{s}-t_{s-1}}\right\}=\varphi_{s}+\psi_{s}x$$

and conditional models as

(A.17) $$f\left\{\frac{Pr(Y_{s}=1|Y_{s-1}=0,X=x,C)}{t_{s}-t_{s-1}}\right\}=\rho_{s}\left(C\right)+\lambda_{s}x,$$

with $Y_{0}\equiv 0$ for convenience.

Recall that our discussion of binary outcomes in Section A.1 started with $p_{1}=E\left\{p_{1}\left(C\right)\right\}$, which follows from the independence of X and C. Even for RCTs, X and C are not in general independent conditional on $Y_{s}$, s>0. This is why rates and hazards are often described as suffering from “in‐built selection bias” (Aalen et al. (2015); Hernán (2010); Martinussen et al. (2018); Sjölander et al. (2015)), and this is also the key reason why effect measures based on rates and hazards can suffer from noncollapsibility even for the identity and log link functions.

To apply Neuhaus's and Jewell's reasoning we must start by removing the conditioning on $Y_{s-1}=0$, as follows:

$$\begin{array}{cc} Pr(Y_{s}=1|X=x,C) & =1-\prod_{k=1}^{s}\left\{1-Pr(Y_{k}=1|Y_{k-1}=0,X=x,C)\right\}\\ & =1-\prod_{k=1}^{s}\left[1-\left(t_{k}-t_{k-1}\right)f^{-1}\left\{\rho_{k}\left(C\right)+\lambda_{k}x\right\}\right]. \end{array}$$

For simplicity, suppose that $t_{1},t_{2},\ldots$ are equally spaced, with $t_{k}-t_{k-1}=\Delta t$, for all k. Also, suppose for now that $\rho_{k}\left(\cdot \right)\equiv \rho \left(\cdot \right)$ and $\lambda_{k}=\lambda$ for all k. Then, writing $p_{x,s}\left(C\right)$ as shorthand for $Pr(Y_{s}=1|X=x,C)$, it can be shown that $p_{1,s}\left(C\right)$ and $p_{0,s}\left(C\right)$ are related via a function $g_{\lambda ,s,\Delta t}$, say, where

$$g_{\lambda ,s,\Delta t}\left(p\right)=1-{\left(1-\Delta tf^{-1}\left[f\left\{\frac{1-{\left(1-p\right)}^{\frac{1}{s}}}{\Delta t}\right\}+\lambda \right]\right)}^{s}$$

and

$$p_{1,s}\left(C\right)=g_{\lambda ,s,\Delta t}\left\{p_{0,s}\left(C\right)\right\}.$$

Since $p_{x,s}\left(C\right)$ is not conditional on $Y_{s-1}=0$, we can apply $p_{1,s}=E\left\{p_{1,s}\left(C\right)\right\}$ as above, where $p_{x,s}=Pr\left(Y_{s}=1|X=x\right)$, and the consequences for (non)collapsibility are as for binary outcomes. We thus turn our attention to the nature of $g_{\lambda ,s,\Delta t}$ for different link functions.

If f is the identity link, the second derivative of $g_{\lambda ,s,\Delta t}$ is:

(A.18) $$g_{\lambda ,s,\Delta t}^{\prime \prime }\left(p\right)=-\lambda \Delta t\left(1-\frac{1}{s}\right){\left(1-p\right)}^{\frac{1}{s}-2}{\left\{{\left(1-p\right)}^{\frac{1}{s}}-\lambda \Delta t\right\}}^{s-2}.$$

This is zero for the first time interval (s=1), but not in general for subsequent time intervals (s≥2). For permissible values of λ, that is, those that correspond to conditional probabilities on the left‐hand side of (A.17) that are between 0 and 1, (A.18) is positive if λ<0, negative if λ>0, and zero if λ=0. However, note that $g_{\lambda ,s,\Delta t}^{\prime \prime }\left(p\right)\to 0$ as Δt→0. Thus, for s≥2, $g_{\lambda ,s,\Delta t}$ is concave for positive λ, convex for negative λ, and linear for λ=0, with the nonlinearity for non‐zero λ disappearing as Δt→0. This is why rate differences are in general noncollapsible, but hazard differences are collapsible. This is illustrated in Figure A.2(a).

If f is the log link, the second derivative of $g_{\lambda ,s,\Delta t}$ is

(A.19) $$g_{\lambda ,s,\Delta t}^{\prime \prime }\left(p\right)=\left(1-e^{\lambda }\right)e^{\lambda }\left(1-\frac{1}{s}\right){\left(1-p\right)}^{\frac{1}{s}-2}{\left[1-e^{\lambda }\left\{1-{\left(1-p\right)}^{\frac{1}{s}}\right\}\right]}^{s-2}.$$

Again, this is zero if s=1, but not in general for s≥2. For permissible values of λ, (A.19) is again positive if λ<0, negative if λ>0, and zero if λ=0. In contrast with the identity link, $g_{\lambda ,s,\Delta t}^{\prime \prime }\left(p\right)$ does not approach 0 as Δt→0. Thus, for s≥2, $g_{\lambda ,s,\Delta t}$ is concave for positive λ, convex for negative λ, and linear for λ=0, with the nonlinearity for non‐zero λ remaining even as Δt→0. This is why both rate ratios and hazard ratios are in general noncollapsible. This is illustrated in Figure A.2(b).

Note that Figure A.2(b) with τ=64 looks very similar to Figure A.1(d); this is because, as τ→∞, and our proportional rates model becomes a PH model, the implied model for the complement of the survival function is indeed a complementary log‐log model.

The consequences of the above for comparing ψ and λ is then as follows. From (A.16), and with the same simplifying assumptions as previously,

$$p_{x,s}=1-{\left\{1-\Delta tf^{-1}\left(\varphi +\psi x\right)\right\}}^{s}$$

and thus,

$$\psi =f\left\{\frac{1-{\left(1-p_{1,s}\right)}^{\frac{1}{s}}}{\Delta t}\right\}-f\left\{\frac{1-{\left(1-p_{0,s}\right)}^{\frac{1}{s}}}{\Delta t}\right\}.$$

For either the identity or log link functions, it then follows that ψ is less than, equal to, or greater than

$$f\left[\frac{1-{\left\{1-g_{\lambda ,s,\Delta t}\left(p_{0,s}\right)\right\}}^{\frac{1}{s}}}{\Delta t}\right]-f\left\{\frac{1-{\left(1-p_{0,s}\right)}^{\frac{1}{s}}}{\Delta t}\right\}=\lambda$$

according as whether $g_{\lambda ,s,\Delta t}$ is concave, linear, or convex, respectively, which gives |ψ|≤λ with equality only at λ=0 and s=1, but with ψ→λ for the identity link as $\Delta_{t}\to 0$, that is, the same attenuation as noted for binary outcomes with nonlinear CCFs.

In fact, the discussion above can be made slightly more general for the situation where Δt→0 if we start from models for hazards instead of models for rates. Consider the following marginal and conditional hazards models:

$$f\left\{\lim_{\Delta t\to 0}\frac{Pr(t\leq T<t+\Delta t|T\geq t,X=x)}{\Delta t}\right\}=\varphi \left(t\right)+\psi \left(t\right)x,$$

$$f\left\{\lim_{\Delta t\to 0}\frac{Pr(t\leq T<t+\Delta t|T\geq t,X=x,C)}{\Delta t}\right\}=\rho \left(t,C\right)+\lambda \left(t\right)x.$$

These imply

$$\begin{array}{cc} Pr(T\leq t|X=x) & =1-\exp\left[-\int_{0}^{t}f^{-1}\left\{\varphi (s)+\psi (s)x\right\}ds\right] \end{array}$$

and

$$\begin{array}{cc} Pr(T\leq t|X=x,C) & =1-\exp\left[-\int_{0}^{t}f^{-1}\left\{\rho (s,C)+\lambda (s)x\right\}ds\right]. \end{array}$$

If f is the identity link then, without further assuming that ρ(t,C) and λ(t) are the same for all t,

$$\begin{array}{cc} Pr(T\leq t|X=1,C) & =1-\exp\left[-\int_{0}^{t}f^{-1}\left\{\rho (s,C)+\lambda (s)\right\}ds\right]\\ & =1-e^{-\Lambda (t)}\left\{1-Pr(T\leq t|X=0,C)\right\}, \end{array}$$

where $\Lambda \left(t\right)=\int_{0}^{t}\lambda \left(s\right)ds$. This follows from the fact that $e^{-p}=1-p+O\left(p^{2}\right)$ as p→0.

Similarly, if f is the log link and λ(s)=λ for all s, it can be shown that

$$\begin{array}{cc} Pr(T\leq t|X=1,C) & =1-\exp\left[-\int_{0}^{t}f^{-1}\left\{\rho (s,C)+\lambda \right\}ds\right]\\ & =1-{\left\{1-Pr(T\leq t|X=0,C)\right\}}^{e^{\lambda }} \end{array}$$

without further assuming that ρ(t,C)≡ρ(C) for all t.

This leads to the following two functions for relating $p_{1,t}\left(C\right)$ to $p_{0,t}\left(C\right)$. For the identity link:

$$g_{\Lambda (t)}\left(p\right)=1-e^{-\Lambda (t)}\left(1-p\right),$$

and for the log link:

$$g_{\lambda }\left(p\right)=1-{\left(1-p\right)}^{e^{\lambda }}.$$

Differentiating twice, we find:

$$g_{\Lambda (t)}^{\prime \prime }\left(p\right)=0$$

for the identity link and

$$g_{\lambda }^{\prime \prime }\left(p\right)=e^{\lambda }\left(1-e^{\lambda }\right){\left(1-p\right)}^{e^{\lambda }-2}$$

for the log link, where the latter is exactly the same as for a complementary log‐log model for a binary outcome.

Thus, $g_{\Lambda (t)}$ is linear for the identity link, and for the log link $g_{\lambda }$ is again concave for positive λ, convex for negative λ and linear for λ=0.

Finally, since

$$\Psi \left(t\right)=\int_{0}^{t}\psi \left(s\right)ds=-log\left(\frac{1-p_{1,t}}{1-p_{0,t}}\right)$$

for the identity link, and

$$\psi =log\left\{\frac{log\left(1-p_{1,t}\right)}{log\left(1-p_{0,t}\right)}\right\}$$

for the log link, and since

$$-log\left\{\frac{1-g_{\Lambda (t)}\left(p_{0,t}\right)}{1-p_{0,t}}\right\}=\Lambda \left(t\right)$$

for the identity link and

$$log\left[\frac{log\left\{1-g_{\lambda }\left(p_{0,t}\right)\right\}}{log\left(1-p_{0,t}\right)}\right]=\lambda$$

for the log link, we have that Ψ(t)=Λ(t) for all t for the identity link and |ψ|≤|λ| for the log link. Thus we have collapsibility of the hazard difference in an additive hazards model and attenuation of the marginal hazard ratio in a PH model, as described earlier, but now with an arbitrary baseline hazard function in both models, and a time‐varying hazard difference for the additive hazards model.
