# Supplementary figures and images for: Making apples from oranges: Comparing noncollapsible effect estimators and their standard errors after adjustment for different covariate sets
Source: Biom J. 2020 Dec 14;63(3):528–57. doi: 10.1002/bimj.201900297 (PMC7986756; doi:10.1002/bimj.201900297)

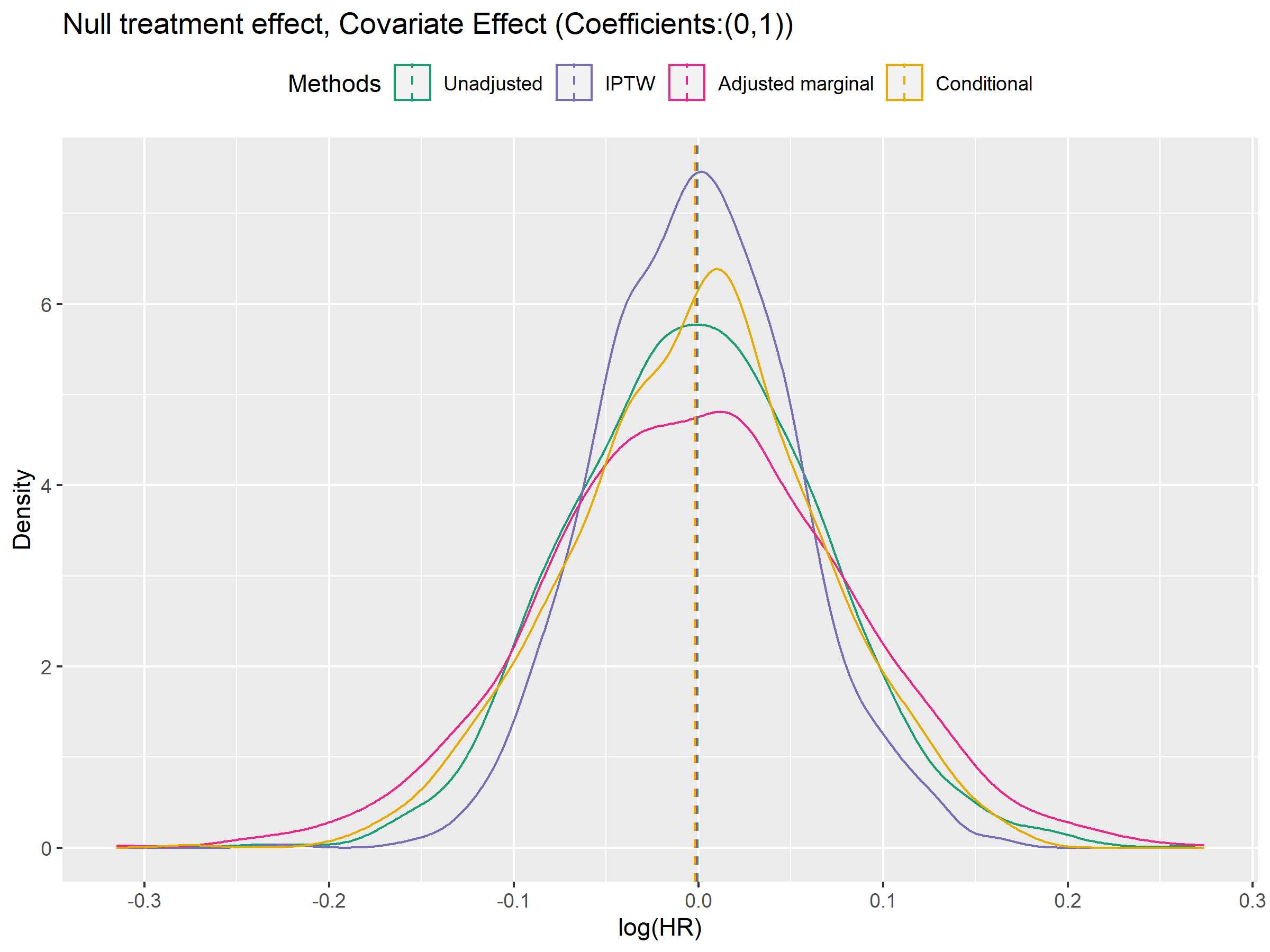

Supplement: Supplementary file 1 — Supporting Information [file BIMJ-63-528-s001.zip › results/HR_Nsim_1000_2m_1000_Coefficient_0_1.tiff]

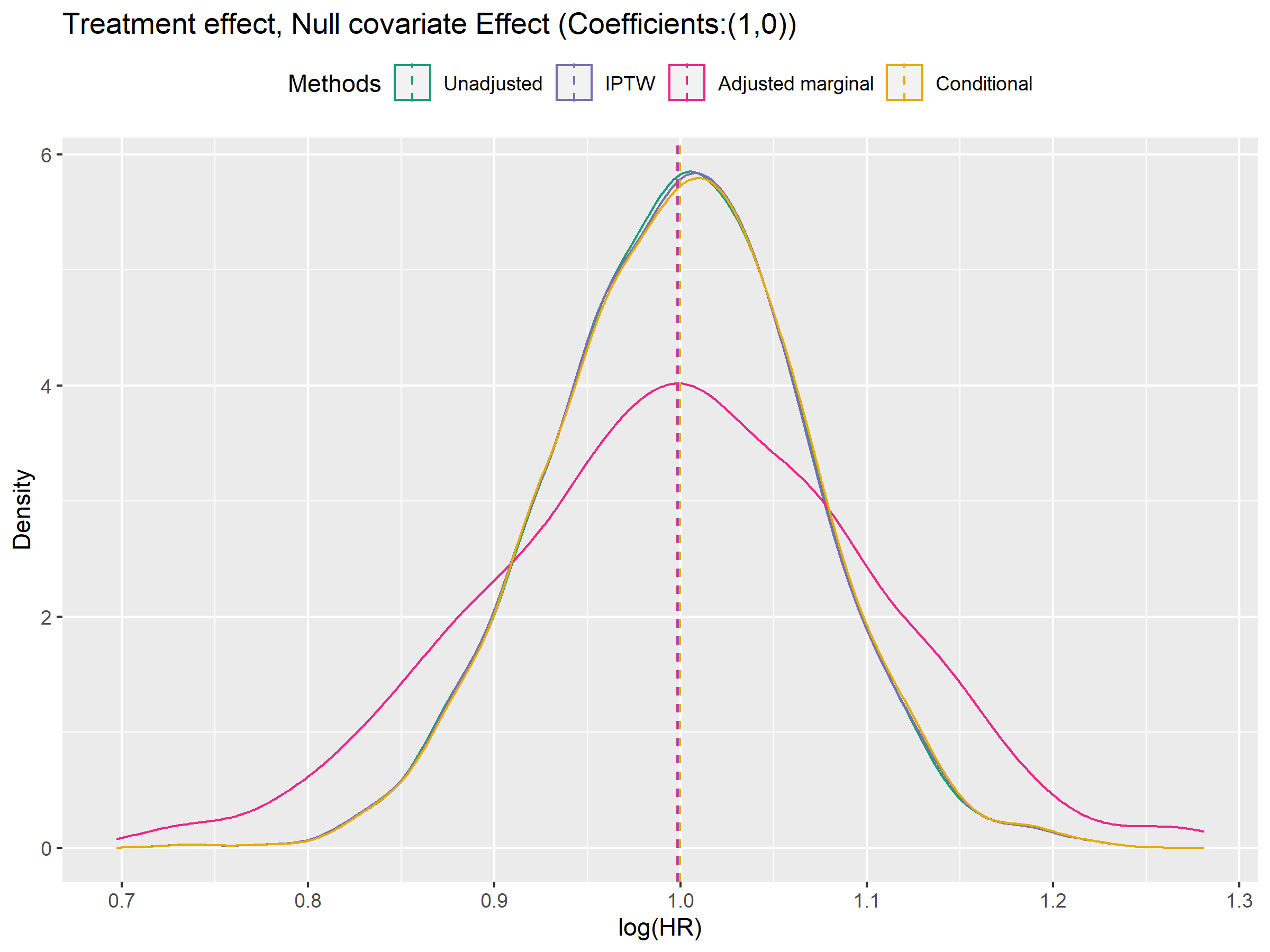

Supplement: Supplementary file 1 — Supporting Information [file BIMJ-63-528-s001.zip › results/HR_Nsim_1000_2m_1000_Coefficient_1_0.tiff]

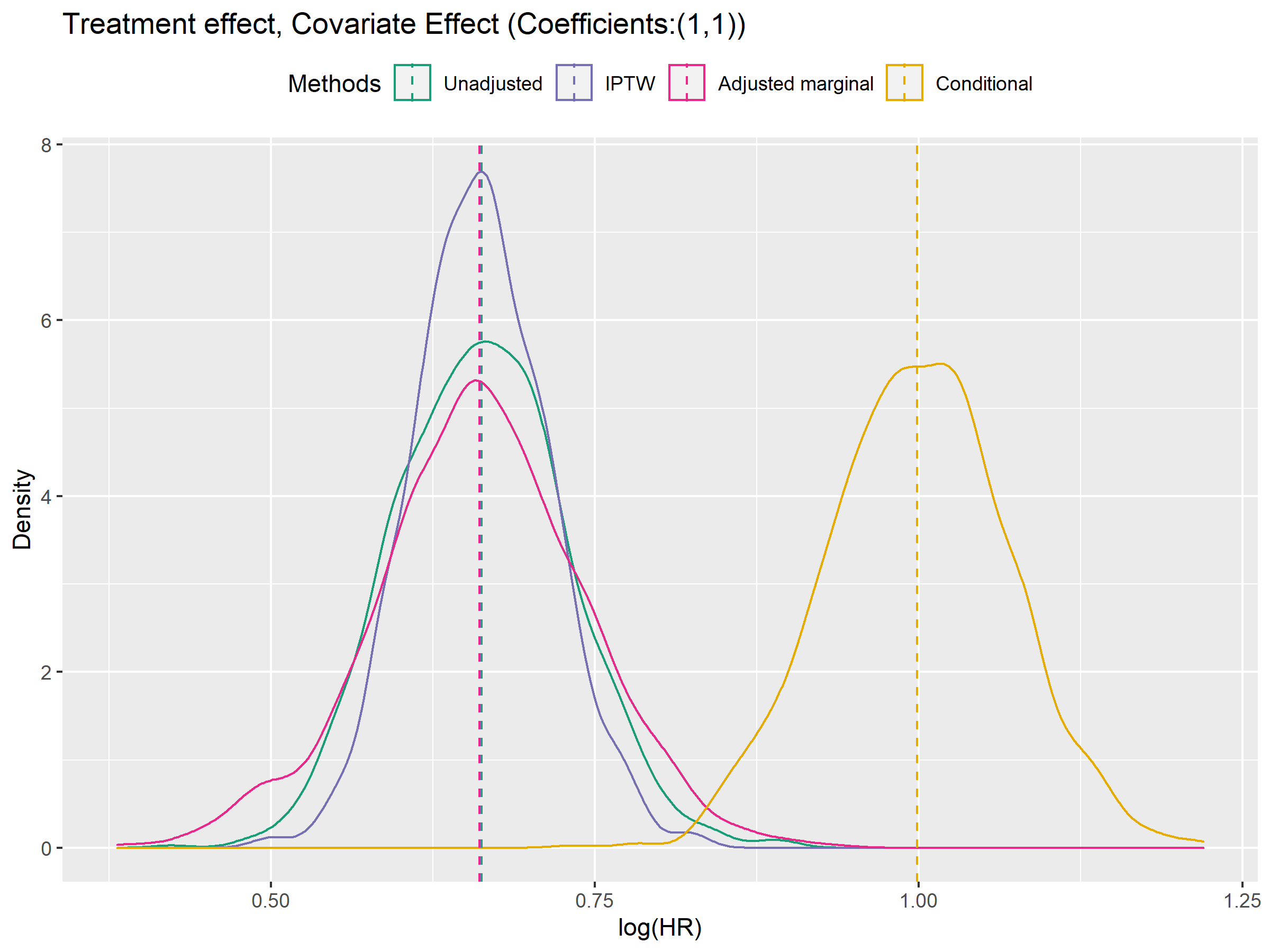

Supplement: Supplementary file 1 — Supporting Information [file BIMJ-63-528-s001.zip › results/HR_Nsim_1000_2m_1000_Coefficient_1_1.tiff]

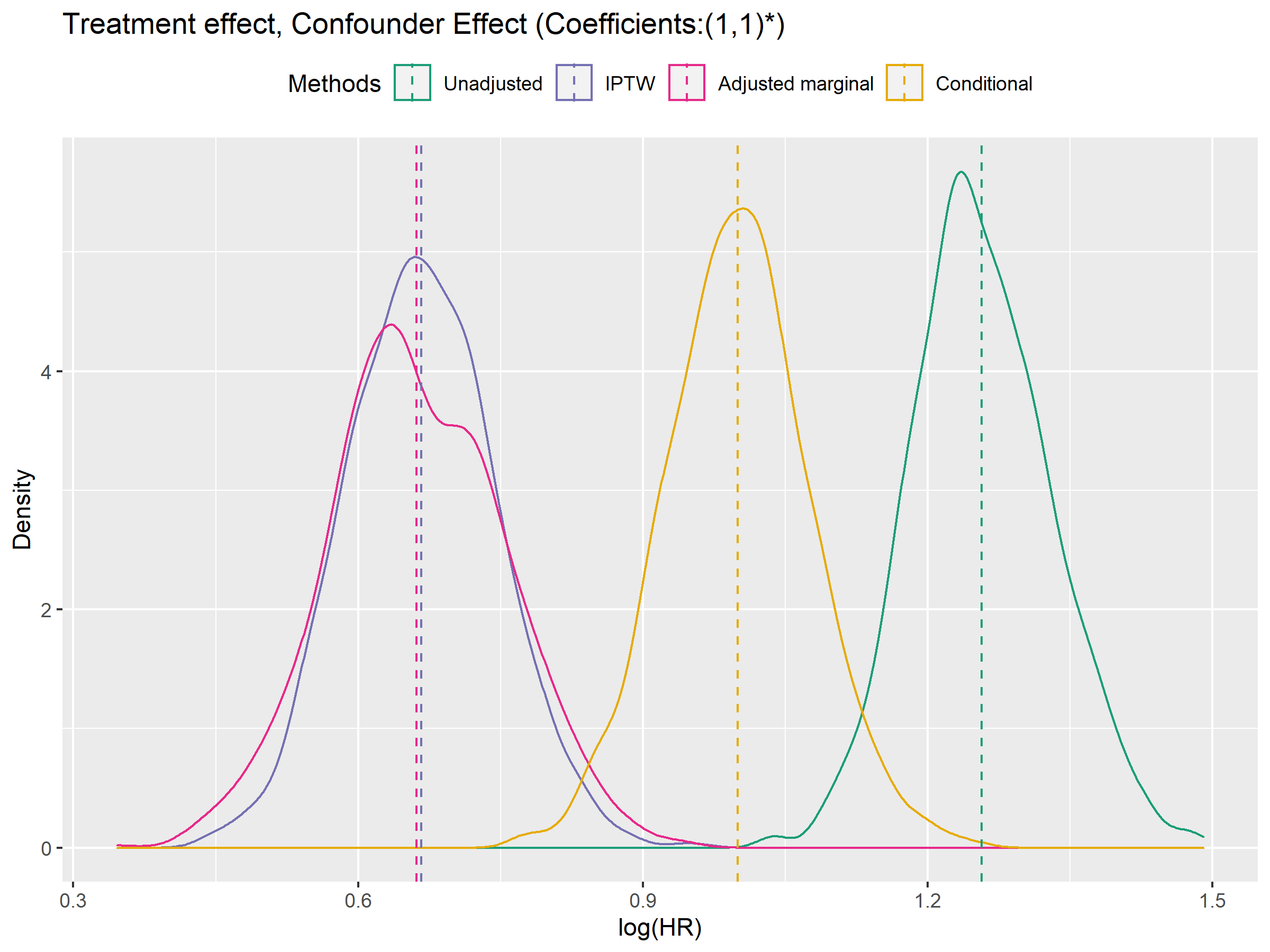

Supplement: Supplementary file 1 — Supporting Information [file BIMJ-63-528-s001.zip › results/HR_Nsim_1000_2m_1000_Coefficient_1_1_confounding.tiff]

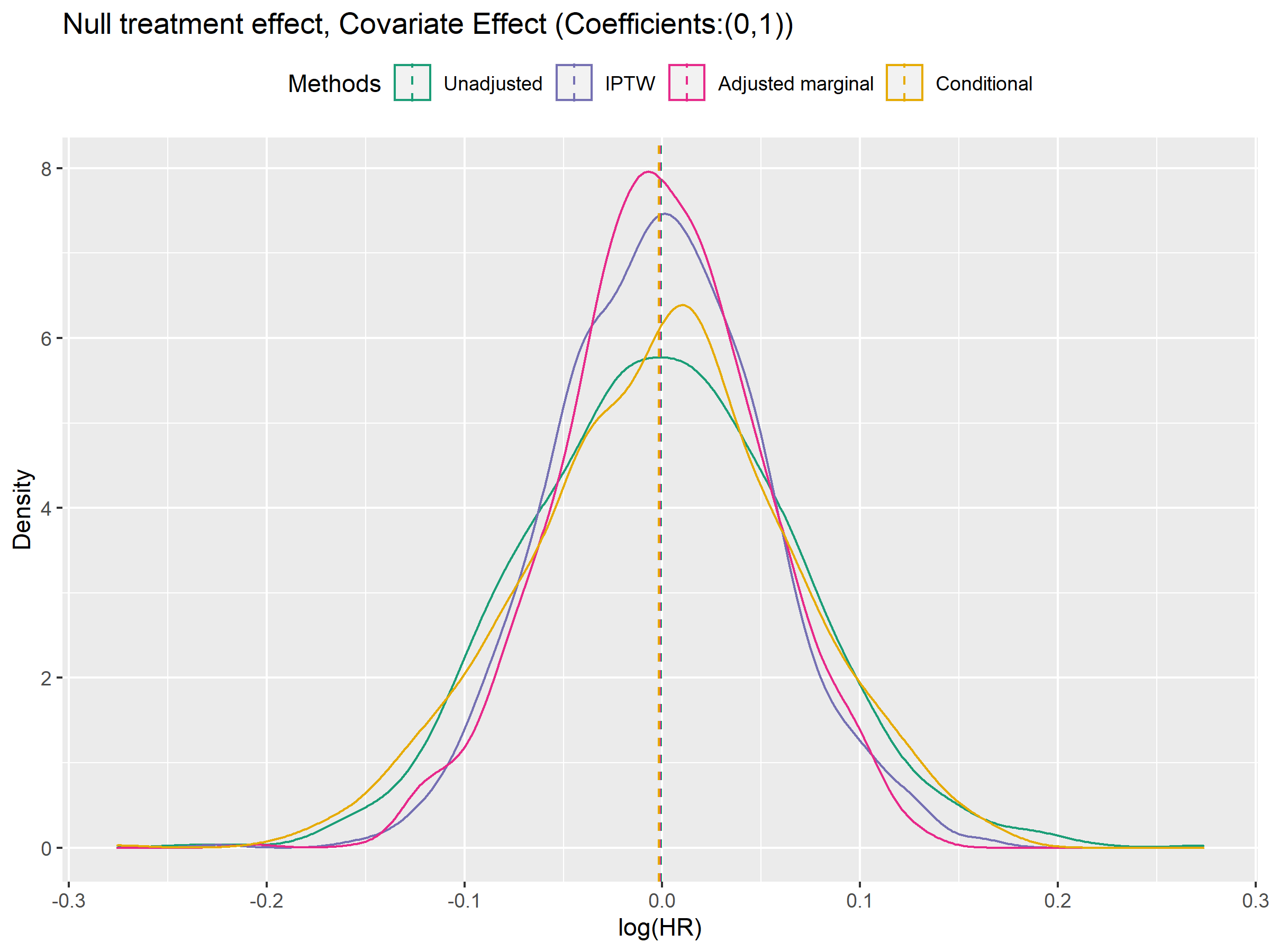

Supplement: Supplementary file 1 — Supporting Information [file BIMJ-63-528-s001.zip › results/HR_Nsim_1000_2m_10000_Coefficient_0_1.tiff]

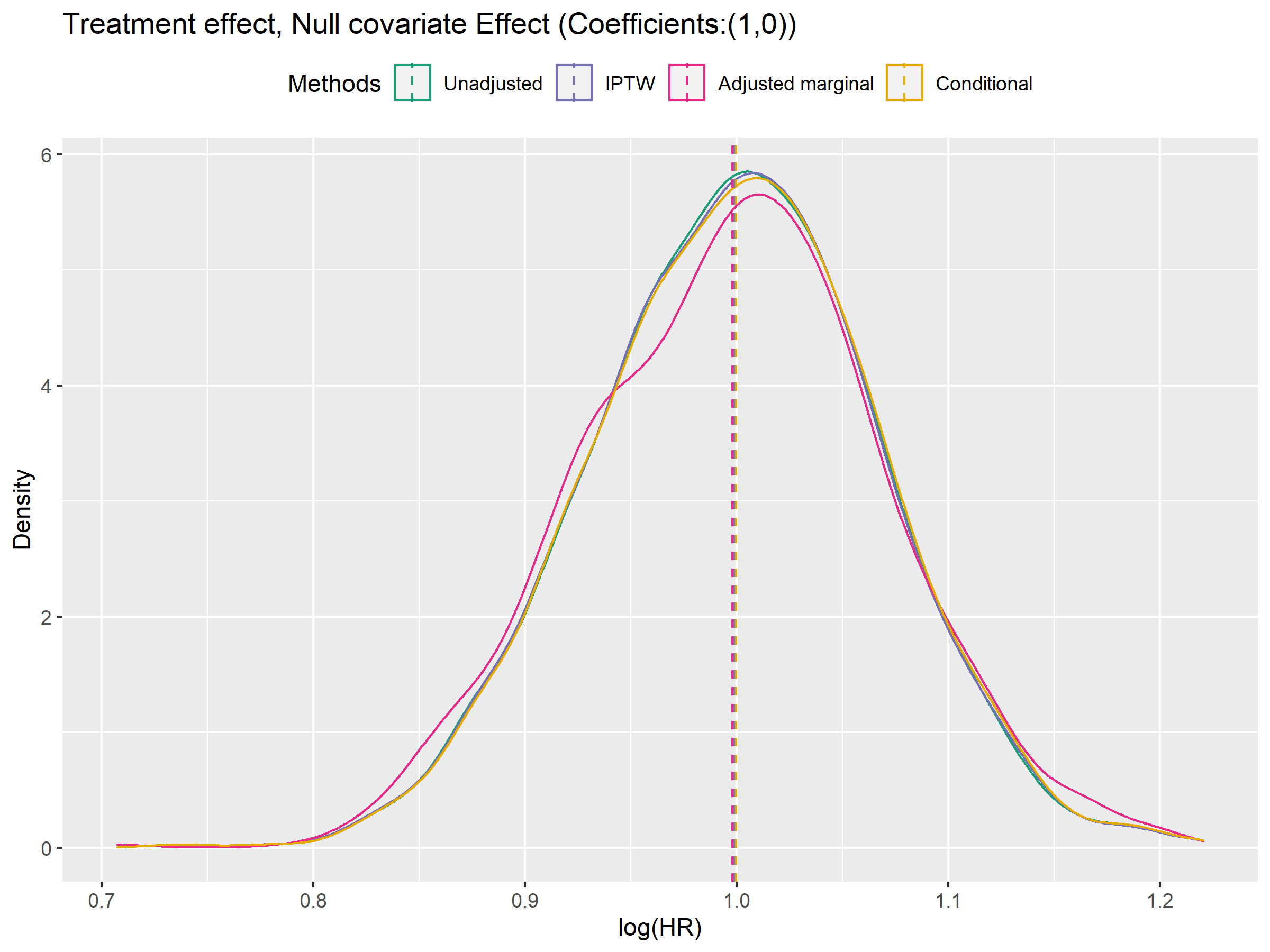

Supplement: Supplementary file 1 — Supporting Information [file BIMJ-63-528-s001.zip › results/HR_Nsim_1000_2m_10000_Coefficient_1_0.tiff]

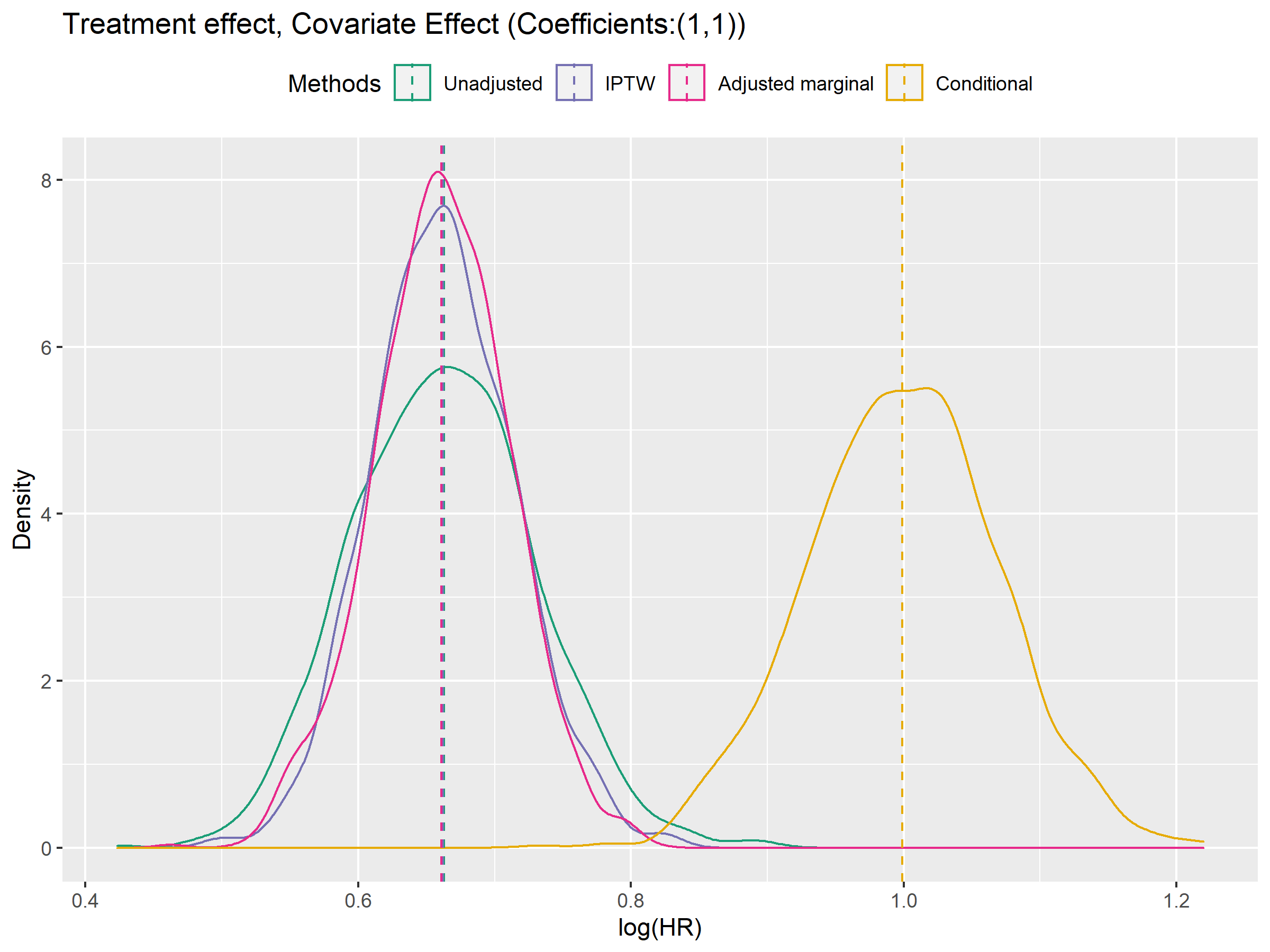

Supplement: Supplementary file 1 — Supporting Information [file BIMJ-63-528-s001.zip › results/HR_Nsim_1000_2m_10000_Coefficient_1_1.tiff]

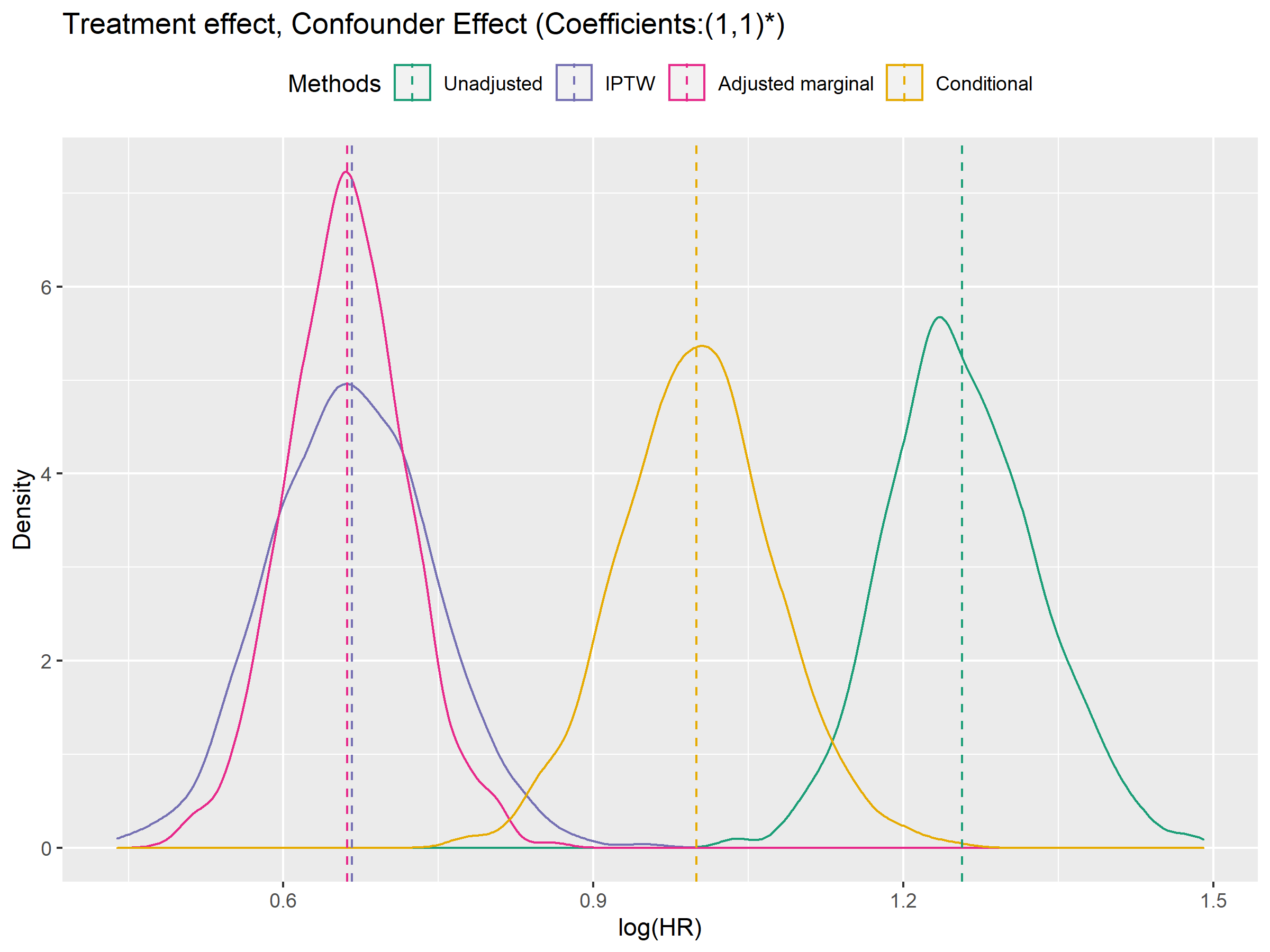

Supplement: Supplementary file 1 — Supporting Information [file BIMJ-63-528-s001.zip › results/HR_Nsim_1000_2m_10000_Coefficient_1_1_confounding.tiff]

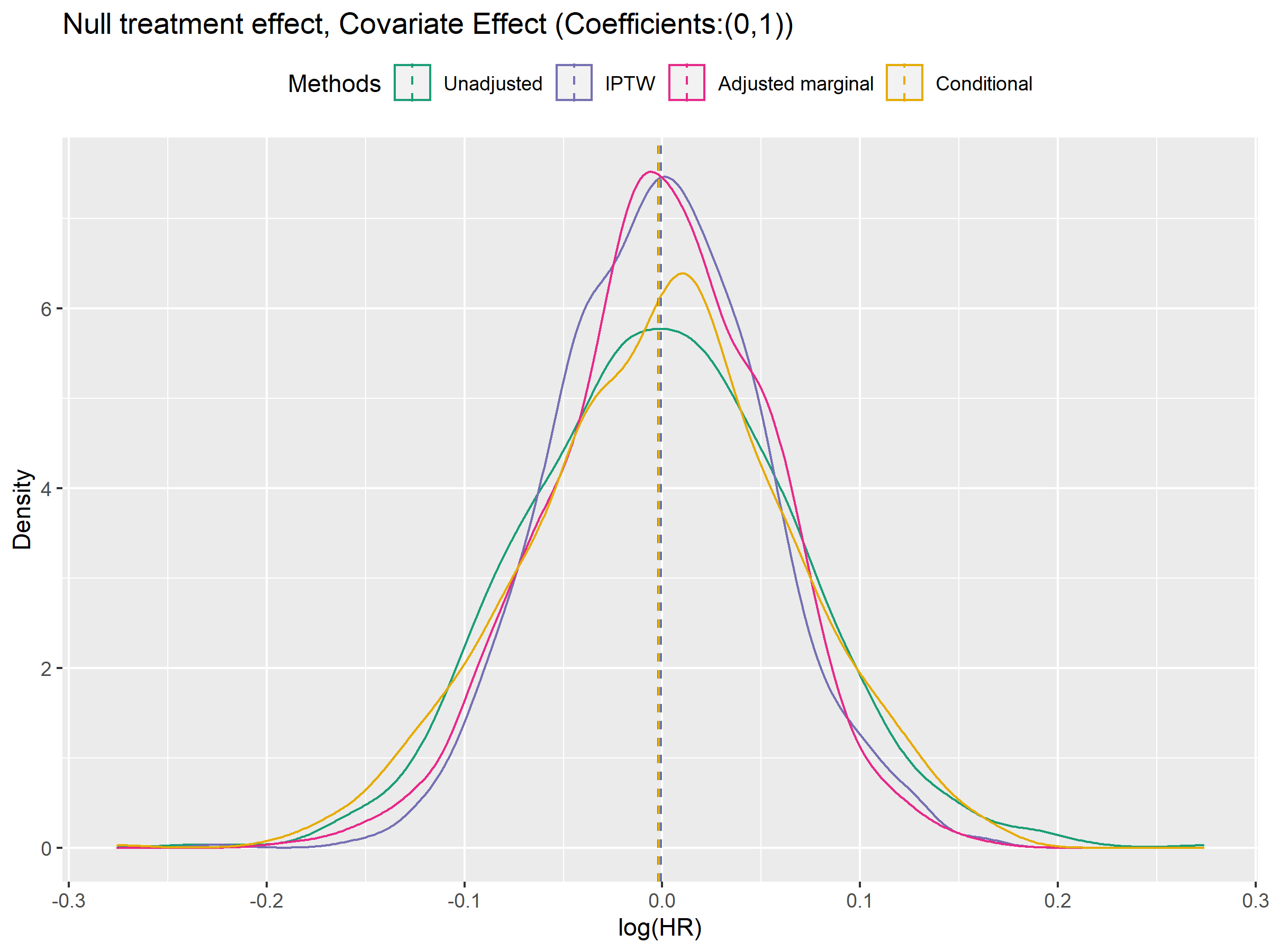

Supplement: Supplementary file 1 — Supporting Information [file BIMJ-63-528-s001.zip › results/HR_Nsim_1000_2m_5000_Coefficient_0_1.tiff]

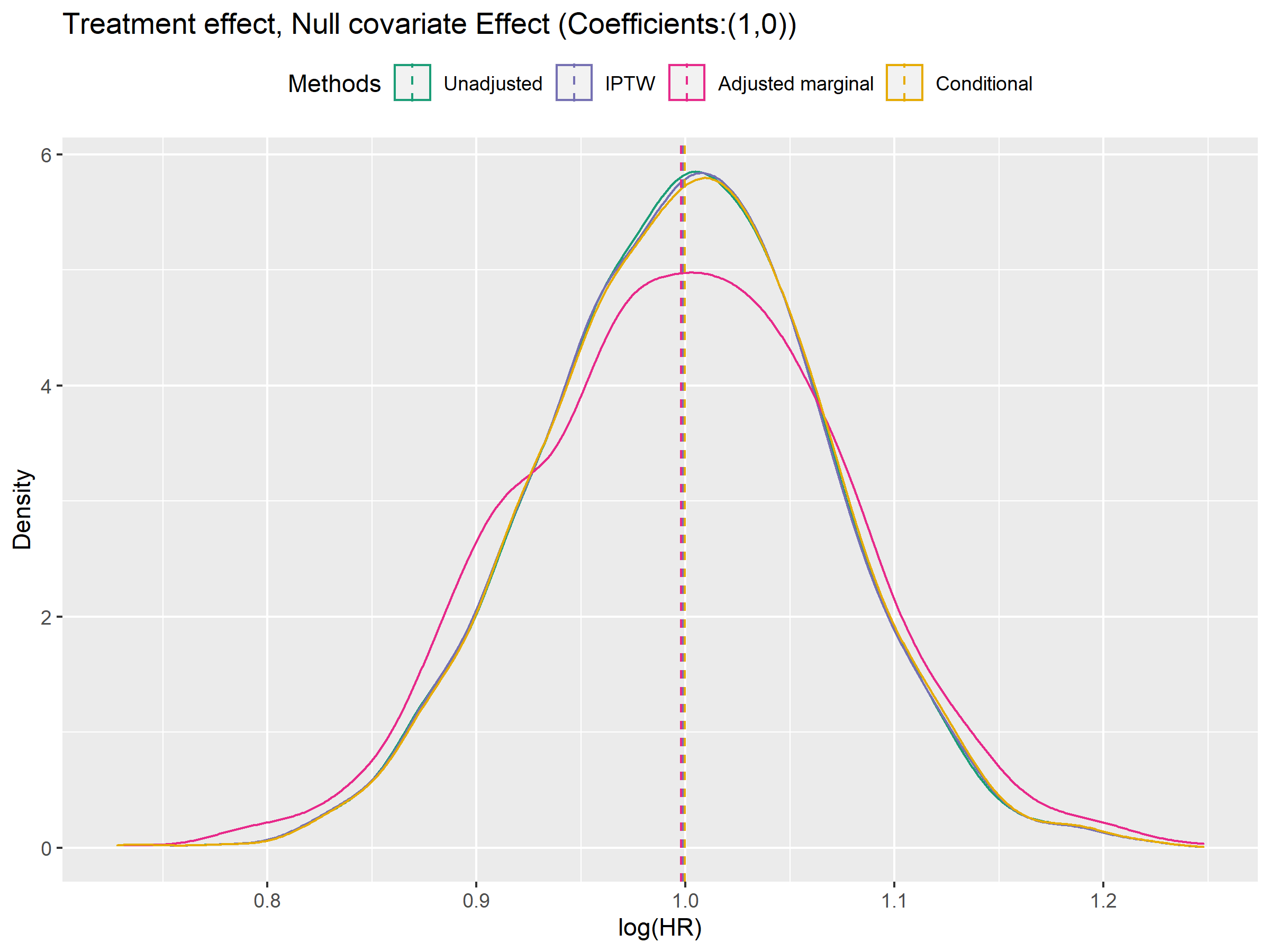

Supplement: Supplementary file 1 — Supporting Information [file BIMJ-63-528-s001.zip › results/HR_Nsim_1000_2m_5000_Coefficient_1_0.tiff]

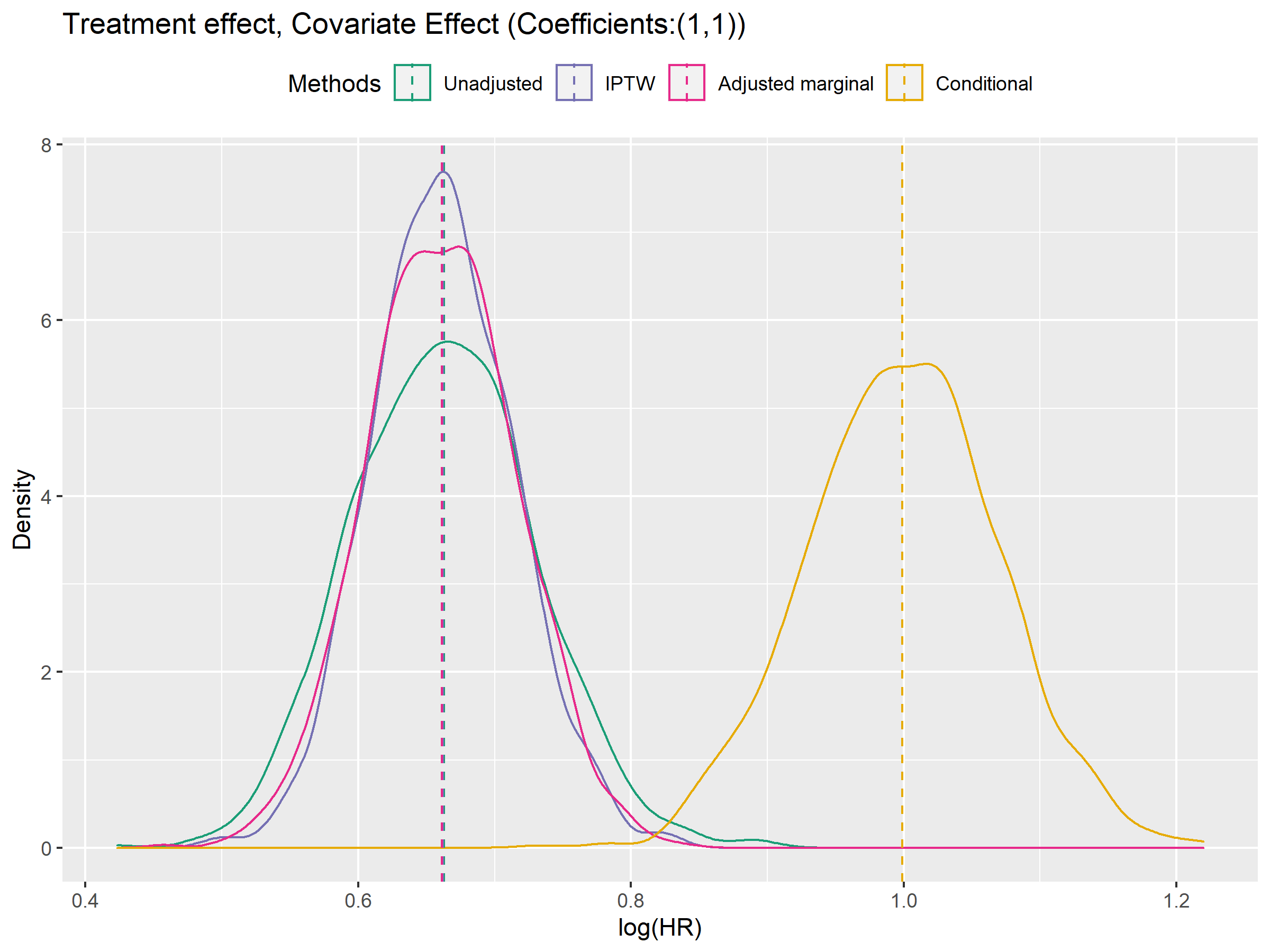

Supplement: Supplementary file 1 — Supporting Information [file BIMJ-63-528-s001.zip › results/HR_Nsim_1000_2m_5000_Coefficient_1_1.tiff]

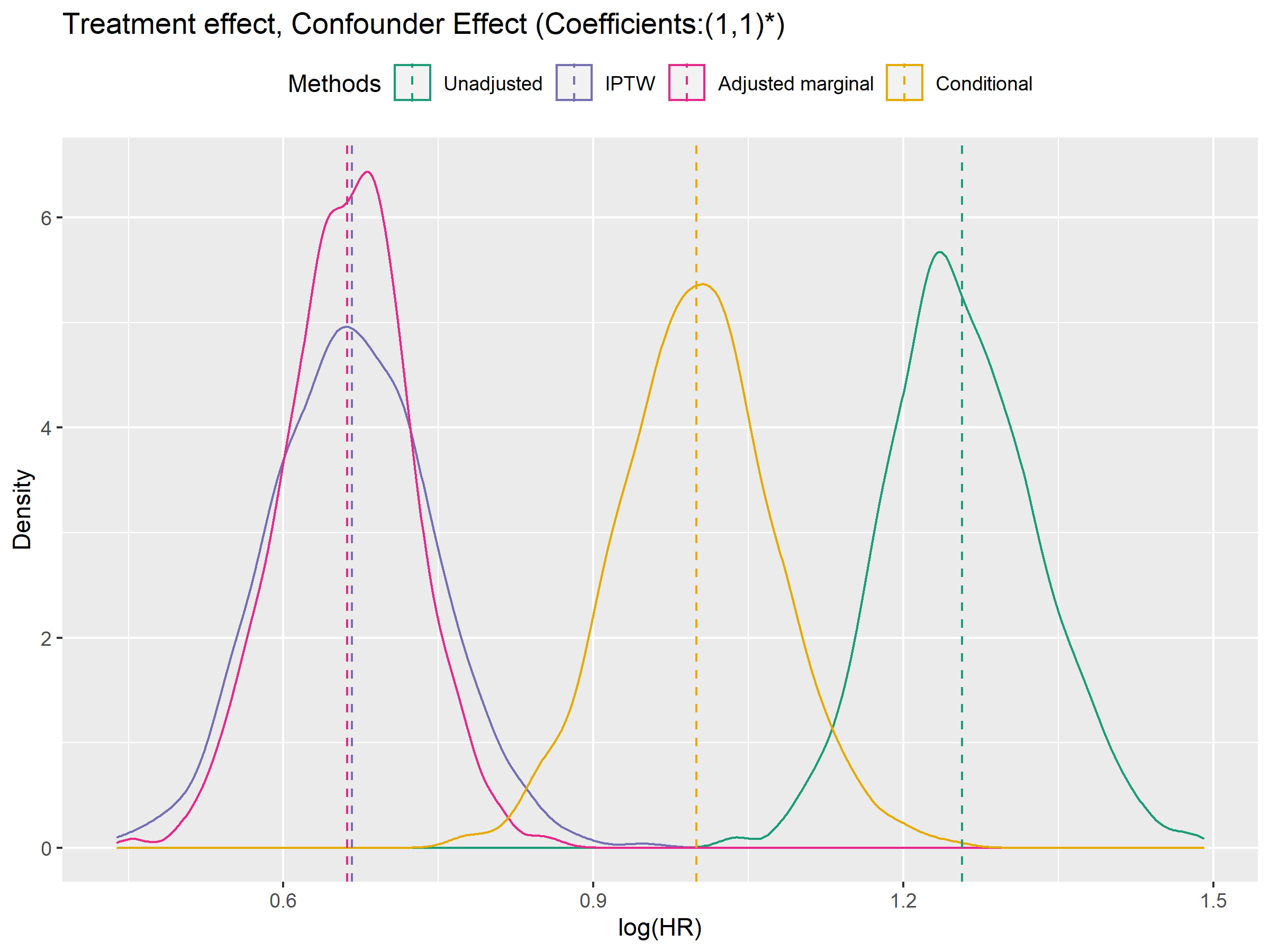

Supplement: Supplementary file 1 — Supporting Information [file BIMJ-63-528-s001.zip › results/HR_Nsim_1000_2m_5000_Coefficient_1_1_confounding.tiff]

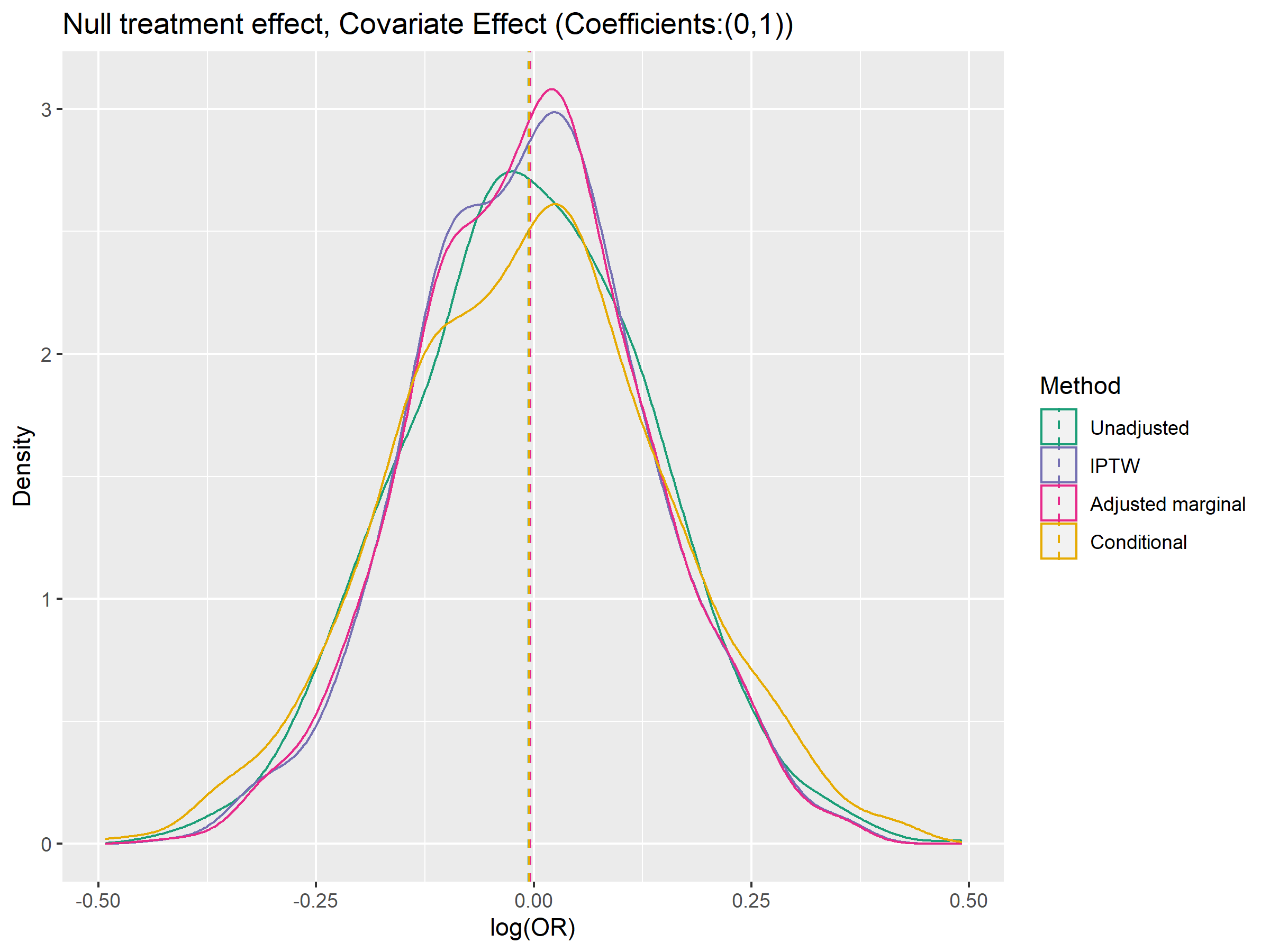

Supplement: Supplementary file 1 — Supporting Information [file BIMJ-63-528-s001.zip › results/OR_Nsim_1000_Coefficient_0_1.tiff]

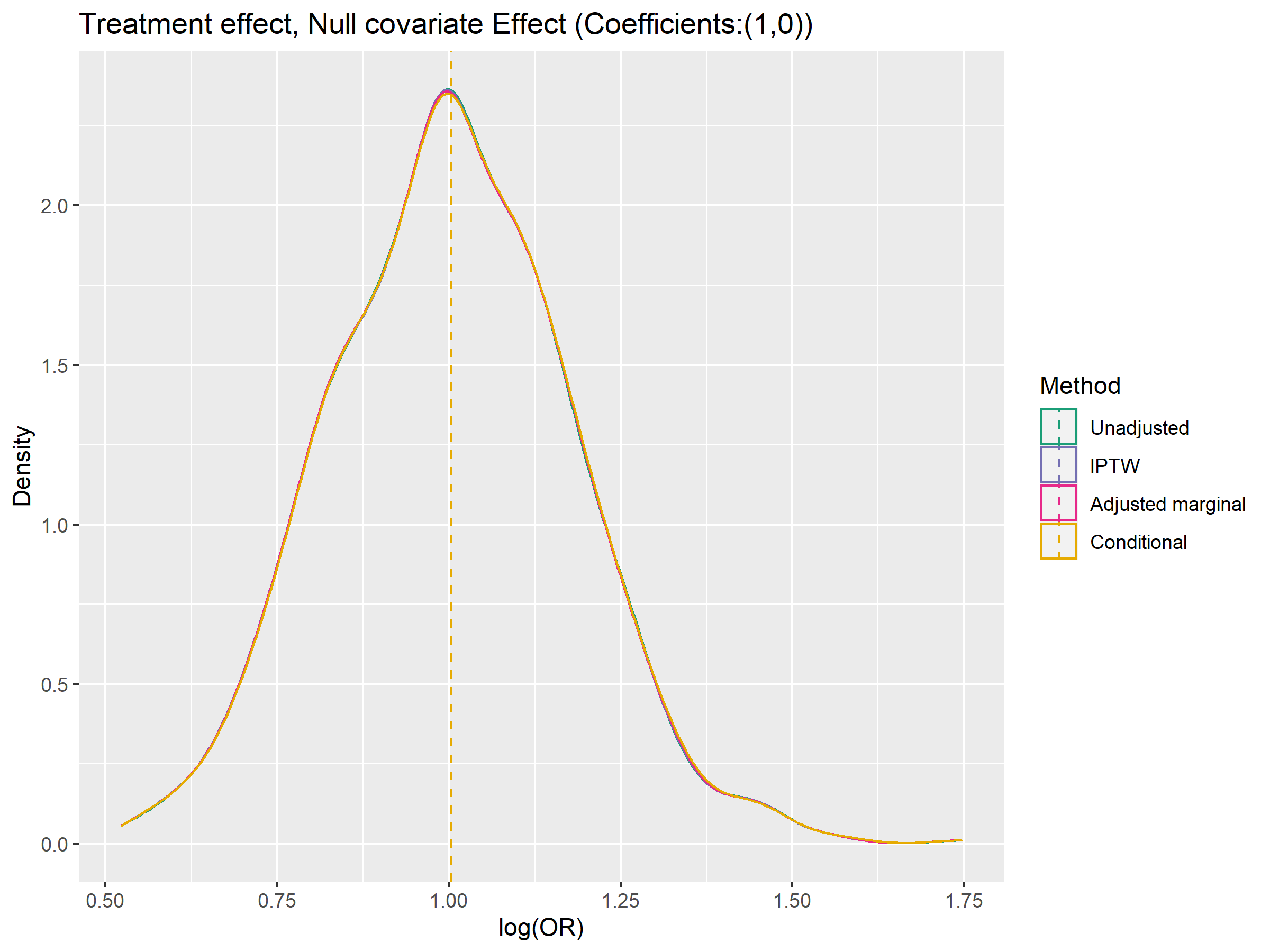

Supplement: Supplementary file 1 — Supporting Information [file BIMJ-63-528-s001.zip › results/OR_Nsim_1000_Coefficient_1_0.tiff]

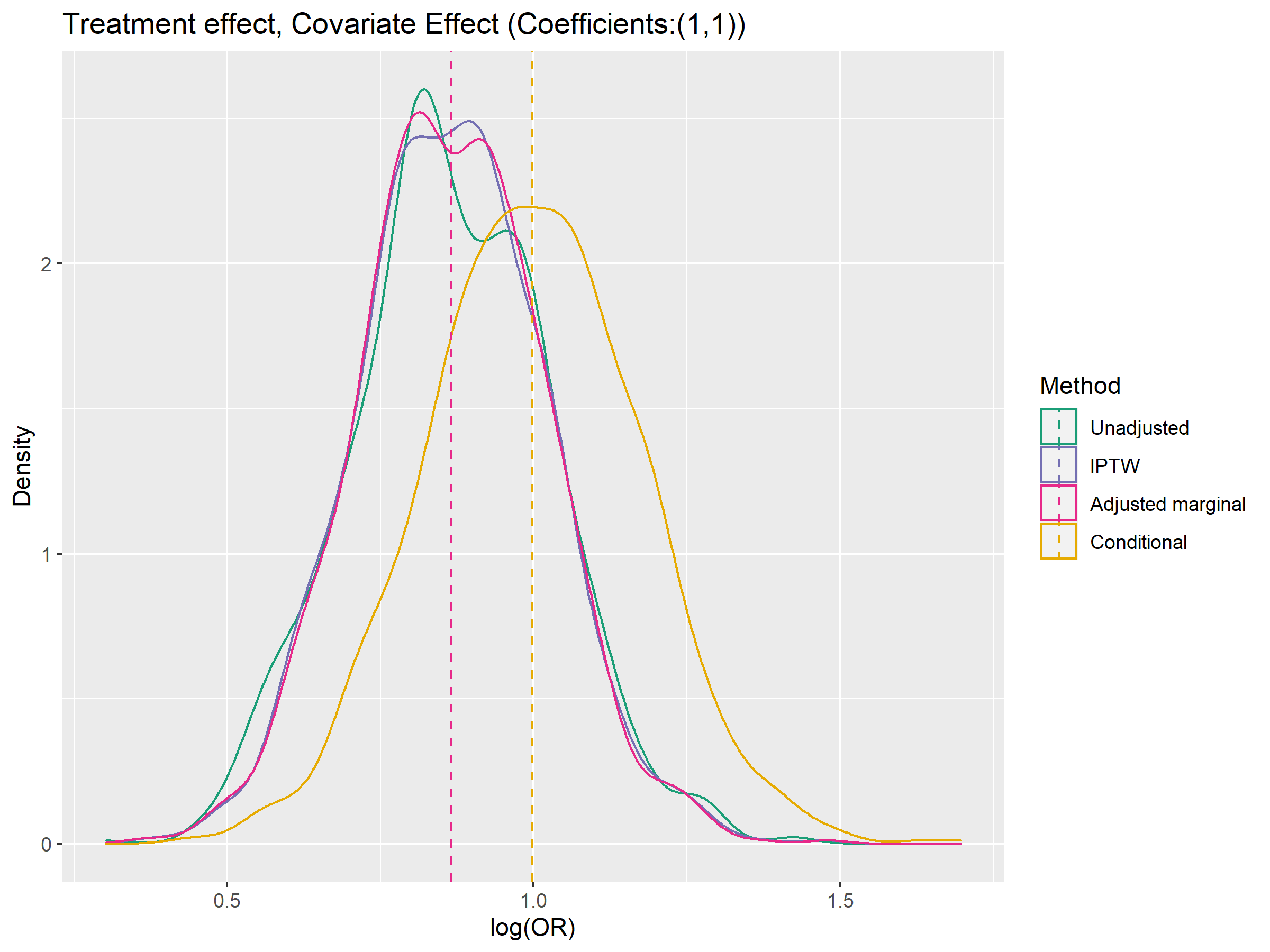

Supplement: Supplementary file 1 — Supporting Information [file BIMJ-63-528-s001.zip › results/OR_Nsim_1000_Coefficient_1_1.tiff]

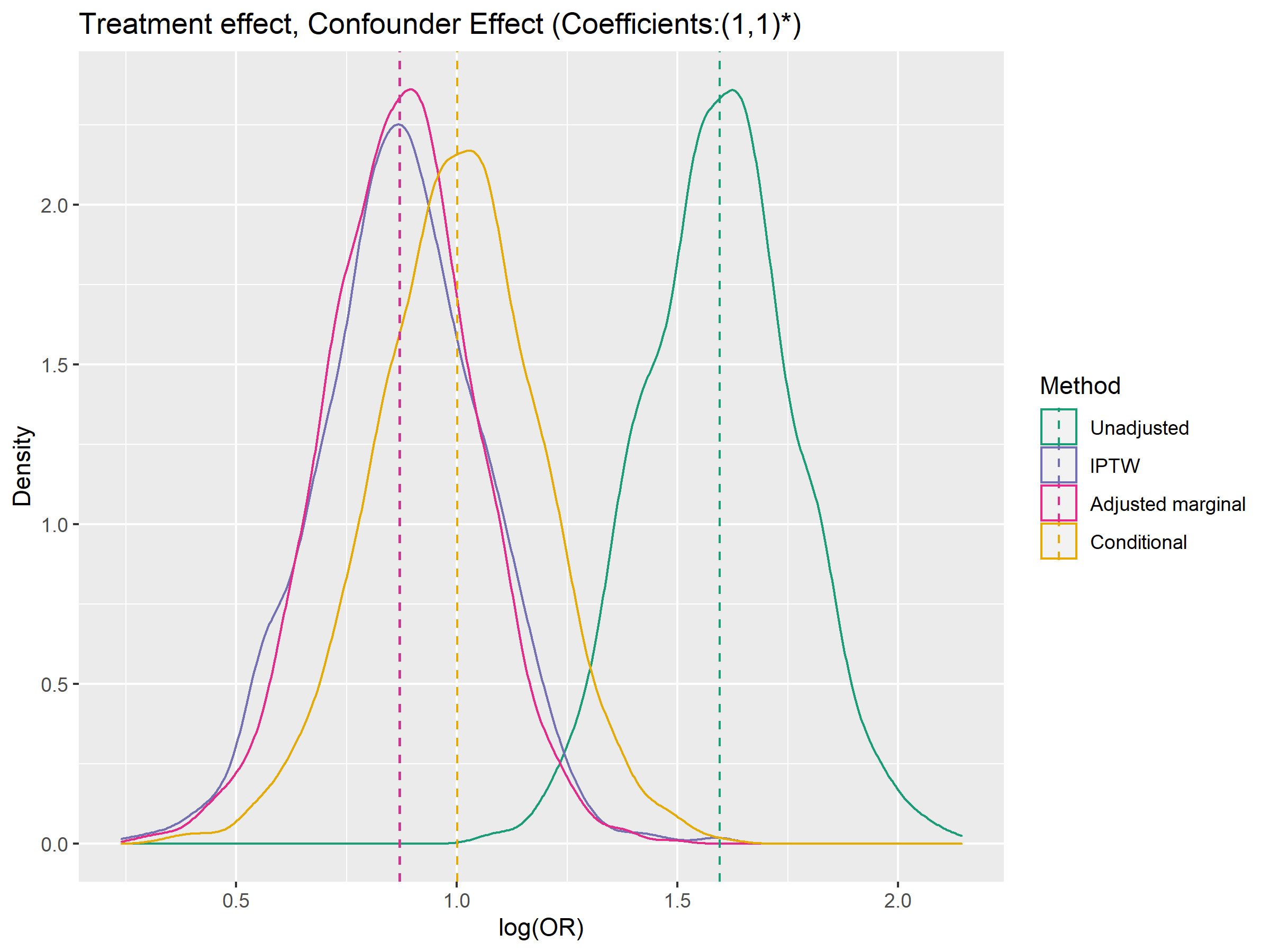

Supplement: Supplementary file 1 — Supporting Information [file BIMJ-63-528-s001.zip › results/OR_Nsim_1000_Coefficient_1_1_confounding.tiff]

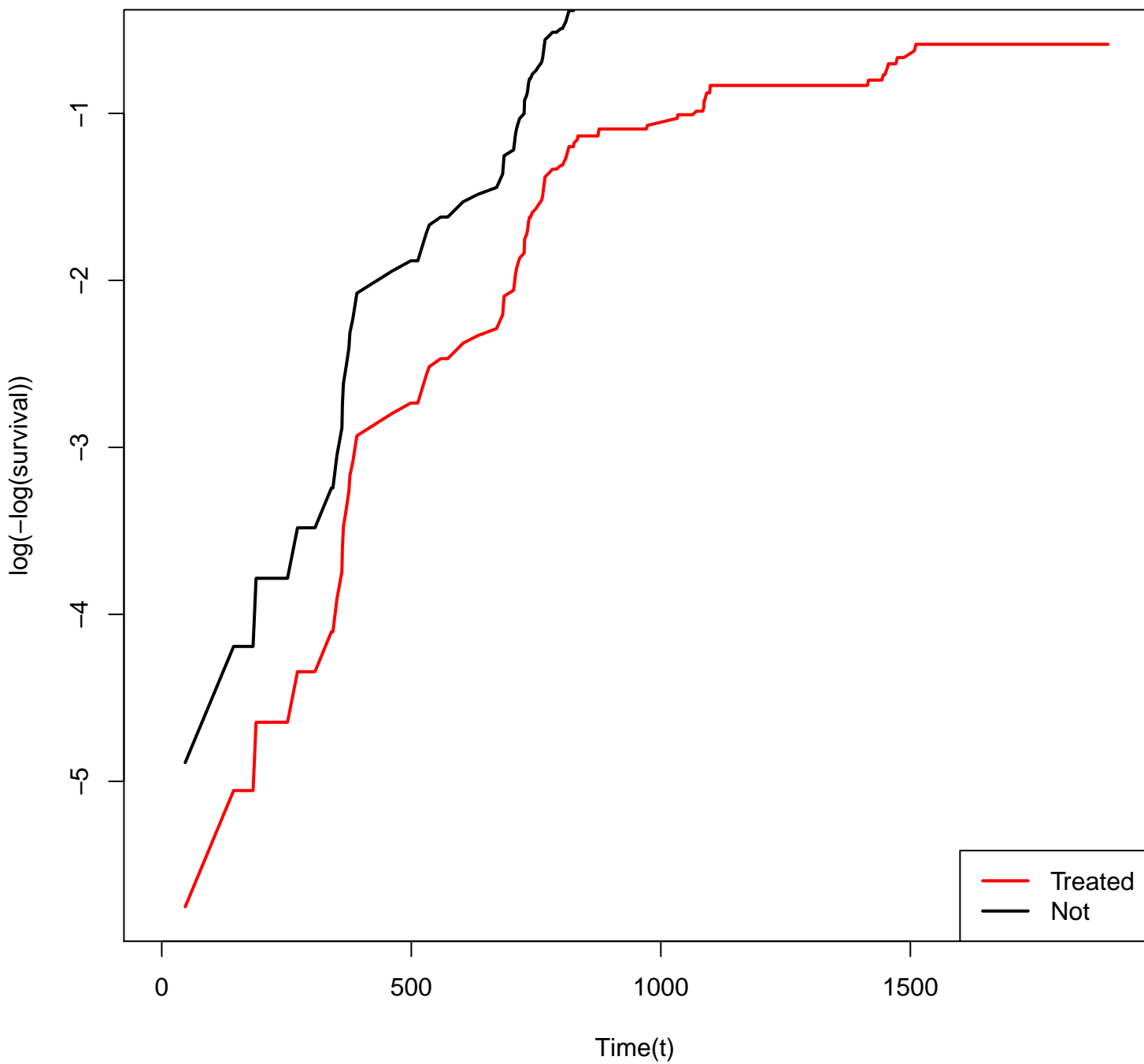

Supplement: Supplementary file 1 — Supporting Information [file BIMJ-63-528-s001.zip › results/pbc_survival_curve.pdf]
